# Beyond linearity - a new Partial Least Squares - Path Modelling (PLS-PM) inner weighting scheme for detecting and approximating nonlinear structural relationships in Structural Equation Models

**DOI:** 10.1371/journal.pone.0345111

**Published:** 2026-03-23

**Authors:** Jorge M. Mendes, Pedro S. Coelho

**Affiliations:** 1 Comprehensive Health Research Centre (CHRC), NOVA Medical School|Faculdade de Ciências Médicas, Universidade Nova de Lisboa, Lisbon, Portugal; 2 Management Information Centre (MagIC), NOVA Information Management School (NOVAIMS), Universidade Nova de Lisboa, Lisbon, Portugal; Universidad Alberto Hurtado, CHILE

## Abstract

A new inner weighting scheme for Partial Least Squares – Path Modelling (PLS-PM) is proposed to detect and approximate nonlinear structural relationships in Structural Equation Models (SEM). PLS-PM is an iterative method used for the estimation of Structural Equation Models (SEM), a widely used analytical tool for assessing causal relationships between latent variables. However, PLS-PM struggles to address the structural nonlinear relationships. To address this limitation, a new PLS-PM inner weighting scheme, *smooth weighting*, is proposed as an additional option to the traditional *centroid*, *factor*, and *path weighting* schemes. A real marketing dataset is used to demonstrate the usefulness of the method for finding evidence of nonlinearity, and a simulated dataset is used to assess its ability to approximate underlying (unknown) nonlinear structural relationships. The results show that the proposed scheme can recover several nonlinear functional forms, outperforming existing inner weighting schemes for commonly used sample sizes (larger than 75 units), regardless of the level of error contamination in the observed manifest variables.

## Introduction

Partial Least Squares – Path Modelling (PLS-PM) is one of the approaches used for estimation [1] of Structural Equation Models (SEM) [2,3], a widely used analytical tool for assessing plausible causal relationships between latent variables in many disciplines, including psychology, biology, management, economics, marketing, and medicine [4–9]. However, PLS-PM, an iterative estimation method, struggles to address structural nonlinear relationships in its inner approximation stage. This study introduces and assesses a new PLS-PM inner weighting scheme to detect and approximate nonlinear structural relationships in structural equation models.

A SEM consists of two models: a measurement model (outer model) and a structural model (inner model). While the former groups manifest variables (observable variables or indicators) into corresponding latent variables (or constructs), the latter comprises a set of regression equations assessing the effects of explanatory latent variables on the dependent latent variables. Over the past 20 years, SEM, also referred to as a second-generation multivariate technique, has gained popularity [5,10,11].

Two approaches exist for estimating relationships in structural equation modelling: the covariance-based SEM (CB-SEM), mainly used for theory confirmation, and the variance-based SEM, also known as PLS-PM, commonly used for predicting causal relationships or confirming measurement (outer) models. Unlike CB-SEM, which uses the maximum likelihood (ML) estimation procedure, PLS-PM is based on ordinary least squares (OLS) and uses observable data (manifest variables) to estimate the structural relationships in the model, minimising the error terms (residual variance) of the endogenous latent variables [12]. In other words, PLS-PM estimates the coefficients of path model relationships by maximising the *R*^2^ values of the target endogenous latent variables.

PLS-PM technique was originally developed by [13] through research devoted to nonlinear iterative least squares, which is the underlying idea of PLS-PM, in which structural equations are specified in linear parametric forms.

In the application framework of SEM and PLS-PM, it is common to find relationships between latent variables that are nonlinear in nature (see [14], for an extensive review of the relationships between satisfaction and loyalty used in the marketing literature).

Nevertheless, the nonlinear approaches to SEM are almost exclusively developed in the context of CB-SEM or Bayesian approaches. The former approaches include, for example, the work of [15], who describe a procedure to estimate the nonlinear and interactive effects of latent variables in structural equation models, given that the latent variables are normally distributed. A semiparametric approach to modelling nonlinear relationships among latent variables using mixtures of linear structural equations was overiewed by [16]. An unconstrained approach to estimate interactions between latent variables through product indicators was proposed by [17]. A general interaction model with multiple latent interaction effects estimated by maximum likelihood via adaptation of an EM algorithm (latent moderated structural equations – LMS) was introduced by [18]. A nonlinear structural equation model where estimation is accomplished via a proposed quasi-maximum likelihood method for simultaneous estimation and testing of multiple nonlinear effects (quasi-maximum likelihood – QML) was introduced by [19]. The LMS [18] and QML [19] estimators with product indicator approaches were compared by [20]. Also, previous work by [21] was extended to the description of a nonlinear structural equation mixture approach that integrates the strength of parametric approaches (specification of the nonlinear functional relationship) and the flexibility of semiparametric structural equation mixture approaches to approximate the nonnormality of latent predictor variables. Nevertheless, all these approaches are limited to the consideration of interactions and quadratic effects. More recently, using a Bayesian approach, attention has been directed to the use of smoothers and additive models as a means to approximate nonlinear relationships between latent variables. Indeed, [22] and [23] developed semiparametric models where the relationships between latent variables are described by an additive structural equation formulated by a series of unspecified smooth functions (Bayesian P-splines).

To the best of the authors' knowledge, with the exception of the work by [24], where four different PLS-PM approaches to estimate quadratic effects are compared, and the work of [25], which uses an ad hoc two-stage methodology to diagnose the functional form of the structural relationships using latent variable scores estimated by the PLS-PM algorithm, no attempt has been made to endow the traditional PLS-PM algorithm with the ability to approximate nonlinear relationships in inner model estimation.

Although it has been suggested that PLS-PM can model nonlinear relationships between structural latent variables [26], the functional forms of the nonlinear relationships between latent variables have to be specified prior to data analysis [24], or the nonlinear nature of the relationship is simply a result of interaction effects [27]. Moreover, strict parametric forms are likely to miss subtle patterns. Customer satisfaction, for instance, could rapidly increase with an initial increase in perceived quality of service but then taper off with a further increase in perceived quality [22].

With advances in statistical methods and computational technology, semiparametric and non-parametric modelling methods based on different smoothing techniques have become more accessible. These methods include smoothing splines and penalised splines [28]. Smoothing offers a valuable alternative to traditional regression techniques. By emphasising patterns and trends in the data rather than fitting complex models, smoothing provides simplicity and interpretability. It excels in capturing the underlying structure of noisy or irregular data, making it particularly useful for exploratory data analysis and visualisation. Smoothing techniques, such as spline smoothing, offer flexibility to adapt to different data shapes and can effectively handle outliers. Moreover, they often require fewer assumptions than regression, reducing the risk of overfitting. Smoothing’s ability to unveil hidden patterns and relationships in data makes it a powerful tool for gaining insight and simplifying complex datasets.

In this paper, we extend the traditional non-parametric PLS-PM algorithm to approximate nonlinear relationships between latent variables by proposing a new weighting scheme for inner approximation. The approximation of the structural relationships of any shape is enabled by the incorporation of additive models [28]. Furthermore, the proposed framework, when used as an exploratory tool, enables researchers to visually examine and interpret the functional relationship between latent variables of interest. Indeed, based on a plot of the smooth fitted functions, a researcher can determine whether the relationship is sufficiently smooth and linear to be captured by a simple linear functional form. More importantly, the proposed framework is compared with PLS-PM using the quality measures that are widely applied by researchers in this field. This assessment is performed with both real and simulated data in a Monte Carlo simulation study.

To the best of the authors' knowledge, no existing work has jointly addressed all the aforementioned issues and proposed a general framework to assess and estimate nonlinear structural relationships in PLS-PM.

The remainder of this paper is organised as follows. In section Material and methods, subsection A new PLS-PM inner weighting scheme: *smooth weighting* describes summarily the traditional PLS-PM approach and its consistent extension. It presents the new inner weighting scheme to incorporate the estimation of nonlinearities in the structural model, the so-called *smooth weighting* scheme. Subsection Data describes the data used in the two empirical illustrations. The actual dataset used in the first example is detailed in subsection Example I: European Customer Satisfaction Index (ECSI) data, and the details of the dataset resulting from a Monte Carlo simulation, including different nonlinear relationships in which the second illustration is based, is described in subsection Example II: a simulated dataset. Details of the software implementation of this algorithm are presented in Software implementation. The results obtained in the first illustration and comparison with those produced by the benchmark software are reported in subsection Example I: European Customer Satisfaction Index (ECSI) data. In subsection Example II: a simulated dataset, the performance of the proposed methodology is assessed against the traditional PLS-PM algorithm, and the results of the bias and root mean square error are detailed. The article ends with the section Discussion where we elaborate on the main findings and results and explain why they matter.

## Materials and methods

### A new PLS-PM inner weighting scheme: *smooth weighting*

The PLS-PM algorithm was originally developed by [13] in 1966 and later extended by [29]. Other detailed descriptions of the stages of the PLS-PM algorithm are provided in [11,30–32].

PLS-PM algorithm is a widely used variance-based method for estimating structural equation models, particularly suited for predictive modelling. The method operates through an iterative algorithm that alternates between estimating latent variable scores, updating outer and inner weights, and computing structural path coefficients. However, its classical formulation imposes a linear structure on the relationships between latent variables, potentially limiting its capacity to capture more complex underlying phenomena.

**Fig 1:**
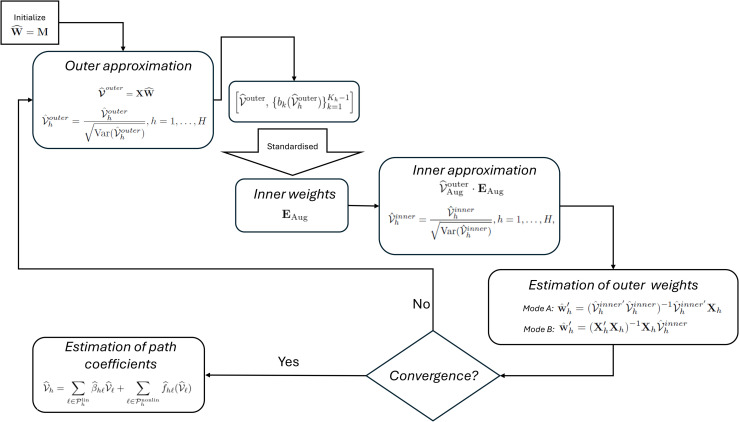
Flowchart for the PLS-PM algorithm.

To address this limitation, we propose a novel smooth weighting scheme that modifies the inner approximation phase of the PLS-PM algorithm. By incorporating nonlinear structural relationships via spline-based additive models [28], our approach – coined **PLSs-PM** – enhances the algorithm’s ability to model smooth, flexible dependencies between latent constructs. The core algorithmic structure of PLS-PM remains intact, but the estimation of latent scores is enriched through the use of cubic regression splines within an augmented representation of latent variable proxies. This formulation is particularly beneficial in models where nonlinear relationships are theoretically expected or empirically observed. The proposed algorithm modifies the classical framework by introducing smooth nonlinear approximations into the inner estimation of latent variable scores. It retains the iterative nature of the original algorithm while extending its flexibility to capture complex dependencies through additive models with cubic regression splines. The core idea is to augment the latent variable proxies with spline transformations wherever a nonlinear structural relationship is specified. This affects steps 1–3 of the classical algorithm. Fig. 1 depicts a diagram describing the main steps of the PLS-PM algorithm. We summarise each step below, but a detailed mathematical formulation and the complete step-by-step derivation of the algorithm are provided in the S1 Appendix.

### Step 1: Outer approximation

Let $𝐗\in {\mathbb{R}}^{n\times P}$ be the data matrix of manifest variables, and let ${𝐖}^{\left(t\right)}\in {\mathbb{R}}^{P\times H}$ be the weight matrix at iteration *t*, structured in blocks per latent variable. The outer proxies of the laten*t* variables are computed as:


$${\hat{V}}^{\text{outer}}=𝐗{𝐖}^{\left(t\right)}.$$ (1)

All manifest variables are assumed to be standardised, and latent proxies are also scaled to have zero mean and unit variance:


$${\hat{V}}_{h}^{\text{outer}}=\frac{{\hat{V}}_{h}^{\text{outer}}}{\sqrt{\text{Var}({\hat{V}}_{h}^{\text{outer}})}},\;h=1,\ldots ,H.$$ (2)

To capture nonlinearities in the structural model, we define an **augmented outer proxy matrix** by applying spline basis expansions to latent variables that enter nonlinear relationships:


$${\hat{V}}_{\text{Aug}}^{\text{outer}}=\left[{\hat{V}}^{\text{outer}},\;\{b_{k}({\hat{V}}_{h}^{\text{outer}}){\}}_{k=1}^{K_{h}-1}\right],$$ (3)

where $b_{k}(\cdot )$ denotes the *k*-th spline basis function applied to the proxy for latent variable *h*, and *K*_*h*_ is the number of knots. These augmented columns are also standardised.

It is worth mentioning that the level of smoothness for the model, determined by the basis dimension *K*, may be crucial. In our implementation of the smooth weighting scheme within the plsEXTplspm package, we rely on penalised regression splines via the mgcv package in R [28]. The selection of the basis dimension *K*, which determines the maximum flexibility of the spline term, is a critical part of the modeling process (more details on the cubic regression spline piecewise basis functions, $b_{k}({\hat{V}}_{ih})$, $k=1,\ldots ,K_{h}-1$, i=1,…,n, h=1,…,H are given in [28]). However, it is important to clarify that in penalised regression spline approaches, *K* does not directly control the effective degrees of freedom (EDF) of the smooth term; instead, it merely sets an upper bound. The actual smoothness is governed by smoothing parameters estimated through Restricted Maximum Likelihood (REML), which penalize excessive flexibility.

Following [28, pp. 242–243]’s recommendations, we selected *K* = 10 as a conservative upper bound that balances flexibility and computational efficiency. This choice aligns with the asymptotic guidance for cubic regression splines estimated via REML, where the basis dimension may grow at a rate of approximately $k=O(n^{1/5})$. For our simulation study, where sample sizes ranged from 75 to 900, this setting is reasonable and consistent with established practice. However, to assess the adequacy of the chosen basis dimension, we integrated into our plsEXTplspm package a diagnostic wrapper function that internally calls gam.check() from the mgcv package. This function performs automatic residual pattern checks by evaluating the correlation between deviance residuals and their nearest neighbours – a method designed to detect under-smoothing and guide potential adjustments to *K*. Specifically, if the estimated EDF approaches the upper limit set by *K* and residual patterns suggest structure not captured by the model, this diagnostic flags the need to increase *K*.

As an additional safeguard, we conducted a brief sensitivity analysis by varying *K* between 8, 10, and 12 in the core simulations. The results showed no substantial change in estimation bias or RMSE, reinforcing that our model is not overly sensitive to this choice within a reasonable range. These findings support the assertion that the default *K* = 10 offers sufficient flexibility for the functional forms evaluated, while avoiding overfitting or computational inefficiency. In conclusion, although the choice of *K* is inherently empirical, our approach combines theoretical justification, automatic diagnostic tools, and sensitivity testing to ensure robustness and transparency in basis dimension selection.

### Step 2: Estimation of inner weights

The inner weights matrix $𝐄\in {\mathbb{R}}^{H\times H}$ in classical PLS-PM is based on adjacency information and one of three schemes: centroid, factorial, or path weighting. In PLSs-PM, we extend the matrix to ${𝐄}_{\text{Aug}}\in {\mathbb{R}}^{H^{*}\times H}$, where $H^{*}=H+\sum_{h}(K_{h}-1)$, to include spline-transformed latent variables.

For a given endogenous variable $V_{h}$, the set of predecessors is split into those assumed to have **linear effects** ${𝒫}_{h}^{\text{lin}}$ and **nonlinear effects** ${𝒫}_{h}^{\text{nonlin}}$. The structural model for $V_{h}$ becomes:


$$V_{h}=\sum_{\ell \in {𝒫}_{h}^{\text{lin}}}\beta_{h\ell }V_{\ell }+\sum_{\ell \in {𝒫}_{h}^{\text{nonlin}}}f_{h\ell }(V_{\ell })+\varepsilon_{h},$$ (4)

where $f_{h\ell }(\cdot )$ is a cubic spline with $K_{\ell }$ knots, represented as:


$$f_{h\ell }(V_{\ell })=\sum_{k=1}^{K_{\ell }-1}\alpha_{h\ell ,k}b_{k}(V_{\ell }).$$ (5)

The weights $\beta_{h\ell }$ and $\alpha_{h\ell ,k}$ are estimated via least squares regression of *V*_*h*_ on its linear and spline-expanded predictors. These estimates populate **E**_Aug_.

### Step 3: Inner approximation of LV scores

The inner proxies are computed as a linear combination of the outer proxies of connected latent variables and their spline transformations:


$${\hat{V}}^{\text{inner}}={\hat{V}}_{\text{Aug}}^{\text{outer}}\cdot {𝐄}_{\text{Aug}}.$$ (6)

These inner proxies are again standardised per latent variable:


$${\hat{V}}_{h}^{\text{inner}}=\frac{{\hat{V}}_{h}^{\text{inner}}}{\sqrt{\text{Var}({\hat{V}}_{h}^{\text{inner}})}}.$$ (7)

This step introduces the **nonlinear influence structure** directly into the latent variable score estimation, improving flexibility without modifying the outer model.

### Step 4: Estimation of outer weights

Outer weights are updated based on Mode A or Mode B weighting [31]:

- **Mode A**: Covariance between indicators and inner proxy,
  $${𝐰}_{h}=\text{Cor}({𝐗}_{h},{\hat{V}}_{h}^{\text{inner}}).$$ (8)
- **Mode B**: Regression of inner proxy on block indicators,
  $${𝐰}_{h}=({𝐗}_{h}^{⊤}{𝐗}_{h}{)}^{-1}{𝐗}_{h}^{⊤}{\hat{V}}_{h}^{\text{inner}}.$$ (9)

The weights are rescaled to ensure identifiability:

- Sum-to-one constraint: $\sum_{p=1}^{P_{h}}w_{ph}=1$, or
- Unit variance constraint: ${𝐰}_{h}^{⊤}{𝐑}_{h}{𝐰}_{h}=1$, with **R**_*h*_ the empirical correlation matrix of **X**_*h*_.

Steps 1–4 are repeated until the maximum relative or absolute change in weights is below a predefined threshold:


$$\max_{h,p}\left|\frac{w_{ph}^{\left(t\right)}-w_{ph}^{\left(t+1\right)}}{w_{ph}^{\left(t+1\right)}}\right|<T_{r}\;\text{or}\;\max_{h,p}\left|w_{ph}^{\left(t\right)}-w_{ph}^{\left(t+1\right)}\right|<T_{a},$$ (10)

with defaults $T_{r}=10^{-7}$, $T_{a}=10^{-3}$.

### Estimation of path Coefficients

Once convergence is reached, In the **smoothing-based hybrid approach**, the structural relationship coefficients $\beta_{h\ell }$ and $\alpha_{h\ell ,k}$ are re-estimated using the factor scores.


$${\hat{V}}_{h}=\sum_{\ell \in {𝒫}_{h}^{\text{lin}}}{\hat{\beta }}_{h\ell }{\hat{V}}_{\ell }+\sum_{\ell \in {𝒫}_{h}^{\text{nonlin}}}{\hat{f}}_{h\ell }({\hat{V}}_{\ell }).$$ (11)

Estimation is conducted using penalised least squares for all the structural relationships involving at least one regression cubic spline. The penalty ensures smoothness and prevents overfitting.

### Data

Assessing the performance of PLS-PM (using the *centroid weighting* scheme) and PLSs-PM algorithms is a critical step. In this context, utilising both an actual dataset and a simulated dataset can provide valuable insights into the efficacy of the two inner weighting approaches.

The PLS-PM algorithm has been widely used for decades to analyse latent variable relationships in structural equation models. However, it may struggle to capture the complex nonlinear relationships that exist in real-world scenarios. To address this limitation, a novel inner weighting scheme, that was specifically designed to accommodate nonlinear relationships among latent variables is proposed.

By applying PLS-PM and PLSs-PM on an actual dataset, we can evaluate their ability to uncover meaningful relationships and assess the model fit. In addition, the use of a simulated dataset allows for controlled experimentation, enabling a direct comparison of the performance of the two schemes under various conditions.

Key performance metrics can be compared between the traditional and new algorithms. This empirical assessment will provide valuable insights into the strengths and weaknesses of each approach and guide researchers in selecting the most appropriate inner weighting scheme for their specific modelling needs. Moreover, leveraging both actual and simulated datasets to evaluate both schemes offers a comprehensive approach to assess their performance and enhances our understanding of their capabilities in handling complex structural equation models, particularly when nonlinear relationships are at play.

The datasets used in this work are available in https://github.com/jmm1972/plsExtpm.

### Example I: European Customer Satisfaction Index (ECSI) data

The European Customer Satisfaction Index (ECSI) is a tool that measures and evaluates customer satisfaction levels across various industries and sectors throughout Europe. Developed to provide a comprehensive understanding of customer experiences, the ECSI plays a pivotal role in helping businesses and policy-makers make informed decisions to improve products and services.

The ECSI assesses customer satisfaction by gathering feedback on various aspects, including product quality, pricing, customer service, and overall experience. This data is then compiled and analysed to generate a satisfaction score for each participating company or organization. These scores enable businesses to benchmark their performance against industry competitors, identify areas for improvement, and devise strategies to enhance customer satisfaction.

The ECSI actual dataset draws in [33]. We used a ECSI banking sector dataset to illustrate the performance of the suggested methodology as it might contain potential nonlinear relationships, particularly with respect to the link between the satisfaction and loyalty latent variables.

**Fig 2:**
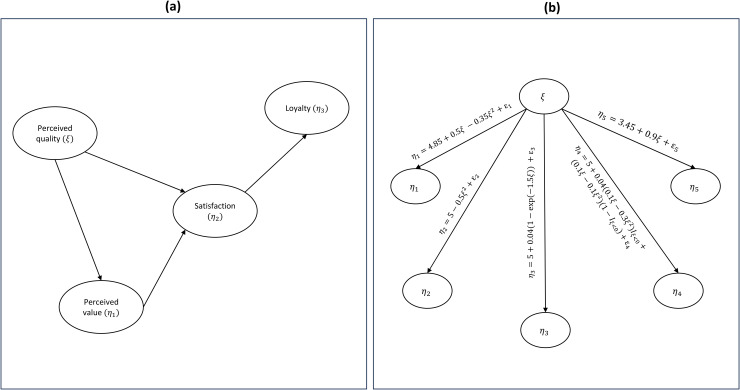
Structural equation models used in examples 1 and 2. **(a)** Structural equation model representing the causes and consequences of customer satisfaction (reduced version of the European Customer Satisfaction Index (ECSI) model). **(b)** Structural model implemented in the Monte Carlo simulation study.

The data are assumed to be generated by the reduced ECSI model depicted in Fig 2(a). The model comprises one formative (Perceived Quality) and three reflective latent variables (Perceived Value, Satisfaction and Loyalty) for the banking sector. S1 Table describes their indicators along with the unidimensionality measures, Cronbach’s alpha (*α*) and Dillon-Goldstein’s rho (*ρ*), for the reflective blocks of manifest variables.

### Example II: a simulated dataset

The second example dataset results from a Monte Carlo procedure in three steps. First, we define the true underlying model. Second, we generated random data from the defined model. Third, given random data, we used the PLS-PM and PLSs-PM algorithms to estimate the model. Finally, the performance of the two algorithms is compared using the absolute bias (B) and the Root Mean Square Error (RMSE). These results were further analysed in terms of algorithmic performance as number of iterations necessary to converge, depending on the level of communality and sample size, and their capability of avoiding negative outer weights for some indicators. Indeed, when a variable possess a very limited number of links to other variables and/or when those links are not of linear nature, the information available by the linear-based inner weighting schemes (e.g., the *centroid* scheme) in the inner approximation is scarce or deviates significantly from the true relationship. This situation may generate negative weights in the outer approximation, especially when the level of shared variance between the latent variable and its indicators (communality) is low.

We define a model consisting of four endogenous latent variables, each of which has a different nonlinear relationship with a exogenous latent variable. The selected functional forms were those used in [25]. For completeness, we decided to add a true linear relationship to assess the extent to which the PLSs-PM can capture these relationships, as a particular case. The equations representing the relationships between latent variables are as follows:


$$\xi_{1}=\text{N}(0,1)$$ (12)

$$g_{1}(\xi )=\eta_{1}=4.85+0.5\xi -0.35\xi^{2}+\epsilon_{1}$$ (13)

$$g_{2}(\xi )=\eta_{2}=5-0.5\xi^{2}+\epsilon_{2}$$ (14)

$$g_{3}(\xi )=\eta_{3}=5+0.04(1-\exp(-1.5\xi ))+\epsilon_{3}$$ (15)

$$g_{4}(\xi )=\eta_{4}=5+(0.1\xi -0.3\xi^{2})I_{\xi <0}+(0.1\xi -0.1\xi^{2})(1-I_{\xi <0})+\epsilon_{4}$$ (16)

$$g_{5}(\xi )=\eta_{5}=3.45+0.9\xi +\epsilon_{5}$$ (17)

where $I_{\xi <0}$ is the indicator function, taking the value 1 if ξ<0, and 0 otherwise, and the vector of disturbances, $\epsilon^{\prime }=c(\epsilon_{1},\epsilon_{2},\epsilon_{3},\epsilon_{4},\epsilon_{5})$ is sampled from a 5-variate normal distribution ${\text{N}}_{5}(0,\Sigma_{\eta })$ where the covariance matrix is


$$\begin{array}{c} \Sigma_{\xi }=(\begin{array}{ccccc} 0.5 & 0 & 0 & 0 & 0\\ 0 & 0.5 & 0 & 0 & 0\\ 0 & 0 & 0.5 & 0 & 0\\ 0 & 0 & 0 & 0.5 & 0\\ 0 & 0 & 0 & 0 & 0.5 \end{array})\; \end{array}$$

**Fig 3:**
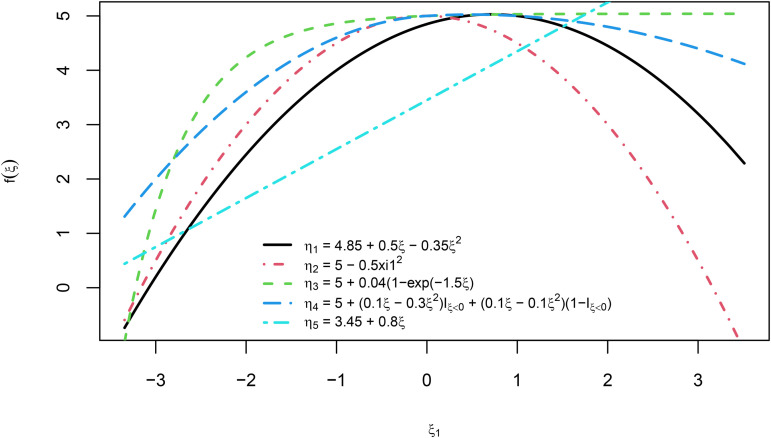
Structural model functional form of the relationships implemented in the Monte Carlo simulation study.

The five functions are visually compared in Fig 3.

To ensure the conditions for the Partial Least Squares consistency, the measurement model has a minimum and fixed number of five indicators per latent variable and unit loadings:


$$x_{l_{\xi }}=\xi +\delta_{\xi },\;l=1,2,3,4,5,$$ (18)

$$x_{l_{\eta_{h}}}=\eta_{h}+\delta_{\eta_{h}},\;h=1,\ldots ,5;\;l=1,2,3,4,5,$$ (19)

where the residual variances, $\delta_{\left(•\right)}$, follows a 5-variate normal distribution ${\text{N}}_{5}(0,\Sigma_{\delta })$ where $\Sigma_{\delta }$ is a diagonal matrix where the residual variance is held constant across indicators and three distinct levels of variance, 3, 1, and 1/3, corresponding to communalities of 25%, 50%, 75%, respectively.

Because the implementation of additive models through regressions splines to approximate nonlinear functional relationships may consume a large number of degrees of freedom, we decided to implement the experiment with different sample sizes ranging from 75 to 900 observations (i.e., 75, 100, 150, 250, 300, 500, 750, and 900). The minimum sample size used, *n* = 75, was imposed such that all models could be estimated using a smoothing spline with *K* = 10 (see the restriction mentioned in A new PLS-PM inner weighting scheme: *smooth weighting* in step 1 of the *smooth weighting* scheme).

Following the described methodology, three populations of 10,000 units were generated (corresponding to the three levels of residual variance). Then, *M* = 1,000 random samples of size *n* = 75, 100, 150, 250, 300, 500, 750, 900 were randomly drawn from the three levels of communality.

A full factorial design (1,000 samples × eight sample sizes × three levels of residual variance) was implemented to capture the eventual interactions at different levels of association between manifest and latent variables and different sample sizes.

Denoting the indices *s* = 1, 2, 3, 4, 5, 6, 7, 8 and *m* = 1, 2, 3 represent the eight different sample sizes and three levels of residual variance, respectively, the absolute bias (B) is defined as:


$${\text{B}}_{sm}\approx \frac{1}{300}\sum_{i=1}^{300}|{\bar{g}}_{h}(\xi_{i})-g_{h}(\xi_{i})|,\;h=1,\ldots ,5,$$ (20)

and root mean square error (RMSE) is given by:


$${\text{RMSE}}_{sm}\approx \sqrt{\frac{1}{M}\sum_{j=1}^{M}\left(\frac{1}{300}\sum_{i=1}^{300}({\hat{g}}_{h}(\xi_{i})-g_{h}(\xi_{i}))\right)},\;h=1,\ldots ,5,$$ (21)

where *M* is the number of Monte Carlo samples, and


$${\bar{g}}_{h}(\xi )=\frac{1}{M}\sum_{i=1}^{M}{\hat{g}}_{h}(\xi_{i}),\;h=2,\ldots ,6$$

is the mean estimated functional relationship between *ξ* and $\eta_{h},h=1,\ldots ,5$, evaluated at a fixed and evenly distributed grid of i=1,…,300 points throughout the range of *ξ*, and $g_{h}(\xi )$ are the known functional relationships given by Eq (13) to Eq (17). All the calculations and figures were done using the estimated final standardised outer proxies (as explained in S1 Appendix) transformed to the original scale using the *theoretical means* (*μ*) and *theoretical standard deviations* (*σ*). Table 1 presents the the empirical means and standard deviation (calculated using the 10 000 generated observations) and the theoretical ones obtained as described in the S1 Text and used to transform the latent simulated latent variables to the original scale.

**Table 1:** Population means and standard deviations of simulated latent variables of example 2 and sample counterparts based on the 10 000 generated observations).

Latent variable	*μ*	$\begin{array}{c} (\bar{x})\; \end{array}$	*σ*	*s*
$\eta_{1}$	4.50	4.49	1.00	0.99
$\eta_{2}$	4.50	4.50	1.00	0.98
$\eta_{3}$	4.92	4.93	0.79	0.77
$\eta_{4}$	4.80	4.80	0.78	0.81
$\eta_{5}$	3.45	3.45	1.14	1.14

### Software implementation

To implement the proposed approach and compare it in terms of performance, it was necessary to implement the PLS-PM algorithm with the three usual inner weighting schemes (as described in S1 Appendix) and extend it to include the **smooth weighting** scheme as described in A new PLS-PM inner weighting scheme: *smooth weighting* and S1 Appendix, using features of package mgcv [28,34–37]. Building on version 1.0–10 (2013) of semPLS package [38], authors changed the code including the *smooth weighting* scheme as an additional option and the estimation of the structural relationships involving cubic regression splines. The resulting set of functions was named plsExtpm. We used R software, version 4.0.5 [39]. The plsExtpm R along with a user manual and all data used in this article are available here https://github.com/jmm1972/plsExtpm.

The correctness of the PLS-PM code was verified by comparing the results obtained with the authors' implementation (using the **centroid weighting** scheme) with the results of the packages plspm [40] and SeminR [41] on the dataset used in Example I. The results were identical, except for some negligible differences owing to rounding inaccuracies. More details about the differences between authors’ implementation, plsExtpm, and plspm and SeminR, regarding ECSI model outer weights, outer loadings, inner Dillon Goldsteins’s *ρ* and R^2^, paths coefficients and total effects can be found in S2 Table, S3, S4 Table, S5 Table and S6 Table.

### Example I: European Customer Satisfaction Index (ECSI) data

The model described in Example I: European Customer Satisfaction Index (ECSI) data and represented in Fig 2(a) was used to illustrate the PLSs-PM algorithm, test the authors' PLS-PM algorithm implementation and highlight the potential of the plsExtpm software.

Table 2 presents the R-squared values of the endogenous latent variables for the PLS-PM and PLSs-PM algorithms (cf. A new PLS-PM inner weighting scheme: *smooth weighting*) (in the case of the latter with (1) all nonlinear relationships with basis dimension *K* = 10 and (2) all linear relationships, except the relationship between Satisfaction and Loyalty that is considered to be nonlinear with basis dimension *K* = 10).

**Table 2:** R^2^ values of the centroid weighting and smooth weighting schemes. R^2^ values are based on correlations between estimated and predicted factor scores.

Latent variable	PLS-PM	PLSs-PM	
	All linear	All nonlinear	Loyalty-Satisfaction nonlinear
Value	60.9	62.1	60.2
Satisfaction	91.3	91.6	91.2
Loyalty	72.2	82.3	82.3

The PLSs-PM algorithm tends to produce larger R-squared values than the PLS-PM algorithm, when all structural relationships are considered to be nonlinear. The difference is more pronounced for the loyalty dependent variable, which is a factor that exhibits a more conspicuous nonlinear relationship with its predictors. These results are consistent with the hypothesis that, by better representing the nonlinear nature of some effects, the PLSs-PM algorithm may offer greater explanatory power than the traditional approaches.

**Fig 4:**
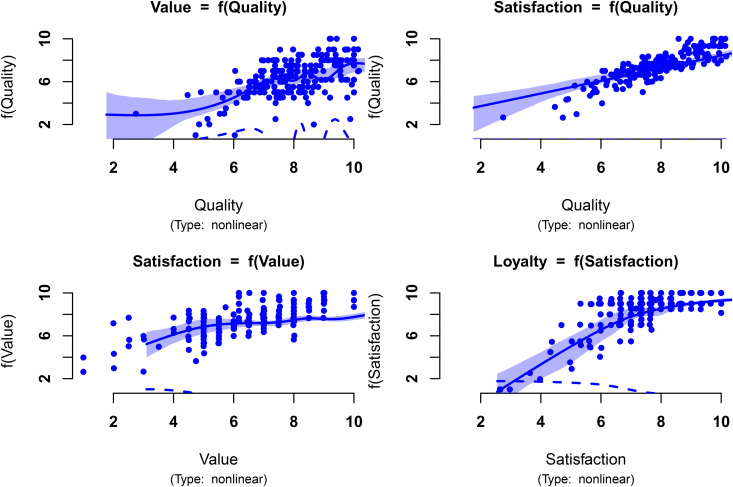
Estimated partial relationships of the reduced ECSI model of Example I, assuming they are all nonlinear and approximated by a cubic regression spline with dimension of the basis *K* = 10. The smooth weighting scheme estimated factor scores are represented as •. The solid blue line corresponds to the estimated relationship, and the area in light blue the corresponding 95% bootstrap credible intervals based on 500 replicates. The first derivative of the partial relationship is represented by the dashed blue line.

Fig 4 illustrates the estimated partial structural relationships assuming they are all nonlinear and approximated by a cubic regression spline (*K* = 10) (check https://github.com/jmm1972/plsExtpm for references on how to plot the partial structural relationships). The plsExtpm contains a function to plot the estimated partial impacts of the structural model and to assess the appropriateness of the used dimension of the basis.

The shape of the estimated factor scores in Fig 4 shows the estimated direct relationship between Perceived Quality and Perceived Value may be described by a linear relationship. The estimated relationship seems to be affected by the leverage effect of some scores on the left side of the plot. Moreover, the partial relationships of Customer Satisfaction (with Perceived Quality and Perceived Value), exhibit also a linear pattern. The figure suggests that the PLSs-PM might generalise to other relationship shapes when a true a linear relationship exists. Therefore, the detailed and patient analysis of these plots are a crucial step for the practitioner to restrict a structural relationship to a linear type when estimatiing the structural model.

Fig 4 also illustrates the estimated direct relationship between Customer Satisfaction Customer Loyalty. The ability to capture nonlinear relationships is particularly noteworthy. Indeed, the PLS-PM algorithm estimates the loyalty vector of scores, as a linear function of the satisfaction scores whereas its PLSs-PM counterpart estimates the loyalty scores as a piecewise linear combination of piecewise basis functions of the satisfaction scores. The additive nature of this model results in a nonlinear relationship, as shown in Fig 4. The gain in the R-squared is significant as Table 2 shows. Furthermore, the manner in which the scores appear in the plot from left to right shows an initial increasing relationship between satisfaction and loyalty. However, their concentration in the top-right corner of the plot hardly suggests a linear relationship over the satisfaction level domain. Indeed, it suggests a decreasing growth rate of the loyalty levels, as first relationship derivative represented by the dashed blue line clearly shows, which is in line with literature mentioned in [22].

**Fig 5:**
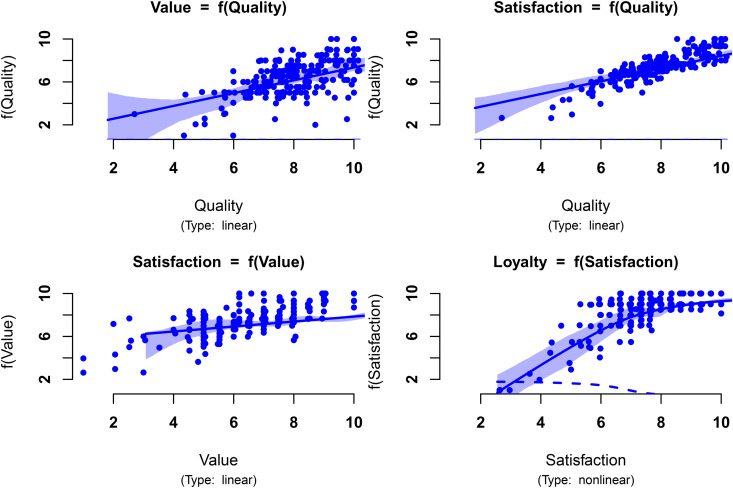
Estimated partial relationships of the reduced ECSI model of Example I, assuming only the relationship between Customer Satisfaction and Customer Loyalty is nonlinear and approximated by a cubic regression spline with dimension of the basis *K* = 10. The smooth weighting scheme estimated factor scores are represented as •. The solid blue line corresponds to the estimated relationship, and the area in light blue the corresponding 95% bootstrap credible intervals based on 500 replicates. The first derivative of the partial relationship is represented by the dashed blue line.

Bearing in mind these conclusion we decided to change the nature of three relationships in the structural model. Changing to linear the first three relationships mentioned in the previous paragraph a second attempt was made. The results are shown in Fig 5. The simplification of the model is the first three relationships does not bring significant losses. Indeed, Table 2 shows the values of the R-Squared are similar to the previous solution, keeping the gain in the explanation of the variance of the Customer Loyalty. Furthermore, one can test whether the basis dimension for a (partial) relationship is adequate as is described in [28] (check https://github.com/jmm1972/plsExtpm for more details). The results of the test indicate the used dimension of the basis, *K* = 10 is not to low. Werther it is to high is not a real problem as long as the sample size is large enough. Indeed, as discussed in A new PLS-PM inner weighting scheme: *smooth weighting* about the trade-off between dimension of the basis and penalisation term shows that even if the dimension increases it is compensated by the penalisation parameter of the penalised least squares method.

In this example, where real data were used, performance was judged by visual inspection of plots describing the relationships and the R-Square values. Indeed, as true population relationships are unknown, it is impossible to assess the estimation accuracy. Consequently, the results of the second illustration comprising the model depicted in Fig 2(b) and the data generated through the Monte Carlo study described in Example II: a simulated dataset are presented in Example II: a simulated dataset, which assesses the performance with respect to the recovery of the functional forms of the relationships, as measured by the absolute bias and root mean square error of the estimators.

### Example II: a simulated dataset

The second experiment aimed to measure the performance of the PLSs-PM algorithm in detecting and approximating the nonlinear relationships between latent variables. As stated earlier, the novel PLSs-PM algorithm does not produce estimates of the parameters of the closed-form structural linear relationships. Instead, it smooths the structural relationships by estimating the coefficients associated with each piecewise linear model in Eq (11). Hence, a direct comparison between the existing and the novel weighting scheme should be accomplished by evaluating their ability to recover structural relationships. Therefore, performance was evaluated with respect to the recovery of the regression function as a whole, as measured by the absolute bias and root mean squared error (RMSE), as described in Example II: a simulated dataset.

The absolute bias (B) and root mean square error (RMSE) of the five relationships for the 24 possible combinations of sample size and residual variance, as described in Example II: a simulated dataset, are presented in the S7 Table.

**Fig 6:**
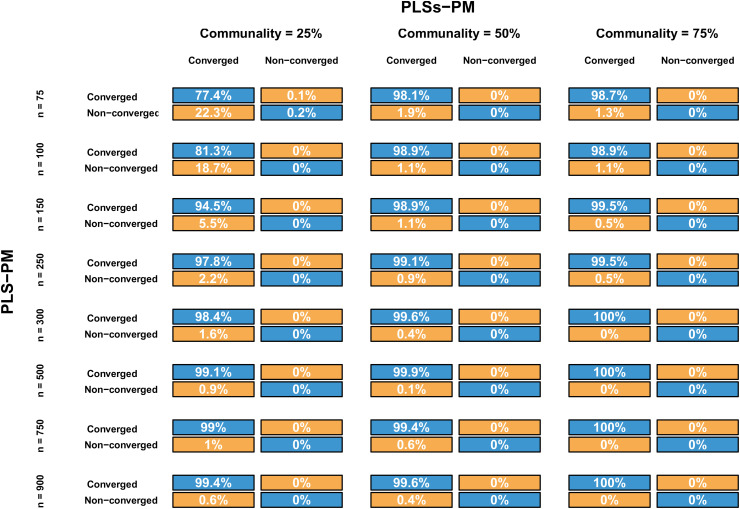
Assessment of the PLS-PM and PLSs-PM algorithms convergence. Each matrix depicts the confusion matrix of *Converged* **and** *Non-converged* iterations, for the same sample, by level of communality and sample size.

**Fig 7:**
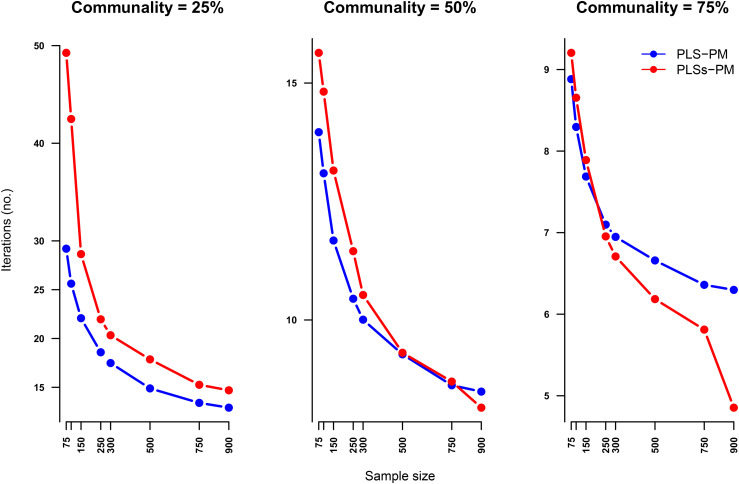
Mean number of iterations to obtain convergence (out of the converged ones), by sample size and communality level. The points in the plots mark, respectively sample sizes of 75, 100, 150, 250, 300, 500, 750 and 900 units. Althought PLS-PM using the *centroid* scheme outperforms PLSs-PM when the level of comunality is very low (25%), when the communality is 50% the latter outperforms the former when sample size is greater or equal than 500 units. And when the communality reaches 75% (which is not in general an hard assumption) it performs better when sample size is gretar or equal than 250 units.

As illustrated in Fig 6 and Fig 7, the PLSs-PM algorithm exhibits a higher convergence failure rate than traditional PLS-PM, particularly when the communality is low (25%) and the sample size is small (e.g., *n* < 300). This behaviour is not unexpected. The introduction of additive models through cubic regression splines in the inner model increases the number of parameters to be estimated, making convergence more sensitive to limited information in the data — a condition accentuated by low communality and small sample sizes. Scientifically, this reflects the fact that the iterative estimation of smoothed structural paths demands a richer latent structure to support the flexible functional forms, and such structure is harder to capture when indicators are weakly related to their latent variables.

To improve convergence under challenging scenarios, we suggest three practical strategies:

1. Increase the sample size, particularly when low communality is unavoidable. Our results show that convergence improves substantially for ≥300, and becomes comparable to PLS-PM for communality larger than 75%.
2. Reduce the basis dimension *K* in spline expansions. Since smoothing penalties limit the effective degrees of freedom, using a smaller *K* (e.g., 6–8) may reduce convergence issues while maintaining sufficient flexibility. This approach is especially useful when the true relationship is expected to be relatively smooth.
3. Apply model simplification heuristics, such as assuming linearity for relationships that exhibit near-linear trends in exploratory analysis. The diagnostic plots generated by our plsEXTplspm package (cf. Fig 4 and Fig 5) can help identify such cases. Alternatively, an initial estimation using linear PLS-PM followed by smooth estimation only on flagged relationships may reduce the model’s complexity and improve convergence.

Finally, our implementation includes verbose logging and diagnostic checks that can alert users to problematic blocks and suggest targeted model simplifications. Future work may explore adaptive estimation strategies that begin with linear approximations and progressively introduce nonlinearity only where justified by the data.

**Fig 8:**
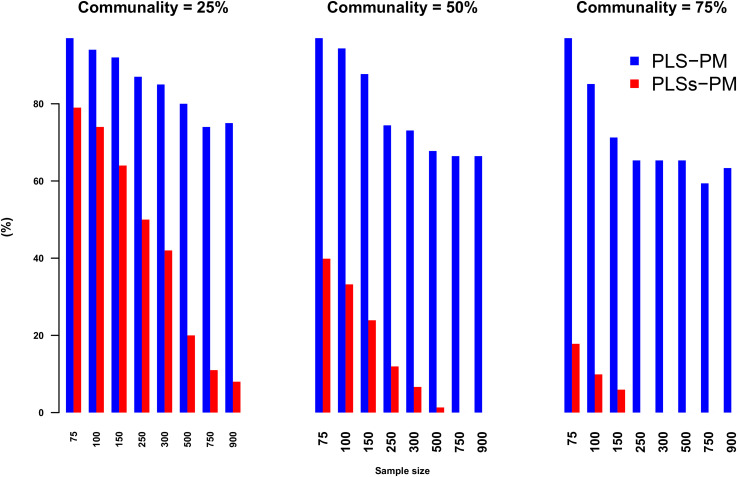
Percentage of samples (out of the converged ones) with with at least one negative outer weight, by sample size and communality level. The percentage of generated outer weights decreases as both the samples size and the communality increase. However, PLSs-PM scheme outperforms PLS-PM in all cases, and it even generates zero negative outer weigths when the communality is 75% and samples size is greater or equal than 300 units.

In Example II: a simulated dataset, the outer approximation of the PLS-PM algorithm may generate negative outer weights for some indicators when a latent variable presents a very limited number of links to other variables and/or when those links are not linear. This likely occurs because either the information generated in the inner approximation might be scarce or deviates significantly from the true nature of the structural relationships. Fig 8 illustrates the performance achieved by PLS-PM and PLSs-PM algorithms in what regards to this matter. Although this problem cannot be completely avoided by the PLSs-PM, it consistently outweighs the PLS-PM.

**Fig 9:**
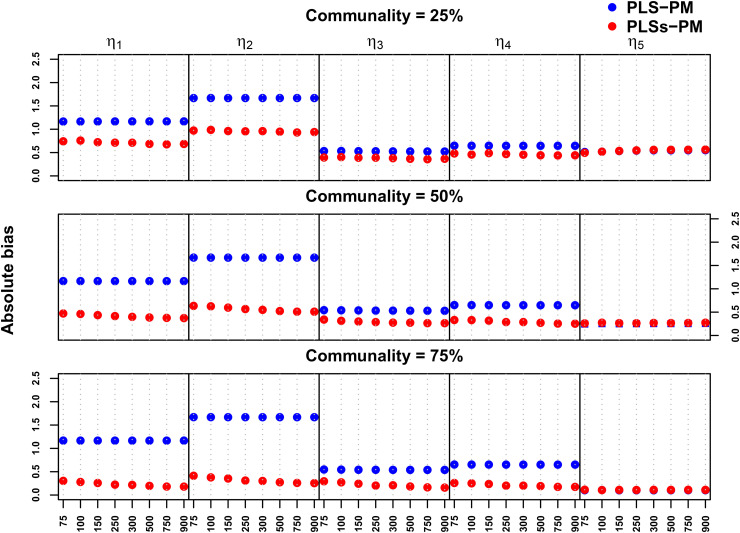
Absolute bias comparison of the five endogenous variables ($\eta_{1},\ldots ,\eta_{5}$) for the eight different sample sizes (n=75,…,900) and three levels of communality (25%, 50% and 75%).

**Fig 10:**
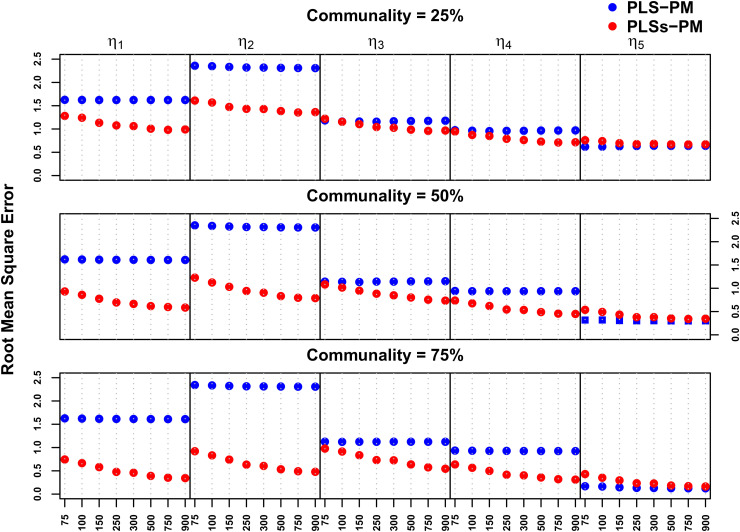
Root mean square error comparison of the five endogenous variables ($\eta_{\text{1}},\ldots ,\eta_{\text{5}}$) for the eight different sample sizes (n=75,…,900 ) and three levels of communality (25%, 50% and 75%).

Fig 9 and Fig 10 compare the accuracy of the PLS-PM and PLSs-PM algorithms on absolute bias and root mean square error regards, respectively.

The absolute bias and mean square error were consistently lower for PLSs-PM than PLS-PM, except in the case of a linear relationship ($\eta_{5}$). Indeed, regarding absolute bias, PLSs-PM algorithm was consistently superior to PLS-PM for the four tested nonlinear forms for sample sizes larger than 75. For the linear relationship $\eta_{5}$, PLSs-PM exhibits similar levels of absolute bias (see Fig 9 and S7 Table). This is not a surprising result after looking at the results of Example I, where _mooth weighting showed its ability to accommodate linear relationships, but simultaneously it can be generalised to other shapes.

The RMSE of PLSs-PM tended to decrease with increasing sample size, leading to progressively increasing precision over PLS-PM. Specifically, PLSs-PM outperformed PLS-PM for nonlinear relationships. An exception occurred in the linear relationship. In this case, its accuracy only gets closer to PLS-PM’s when the sample size increases, even though it happens quickly as the communality levels diminish (see Fig 10 and S7 Table).

**Fig 11:**
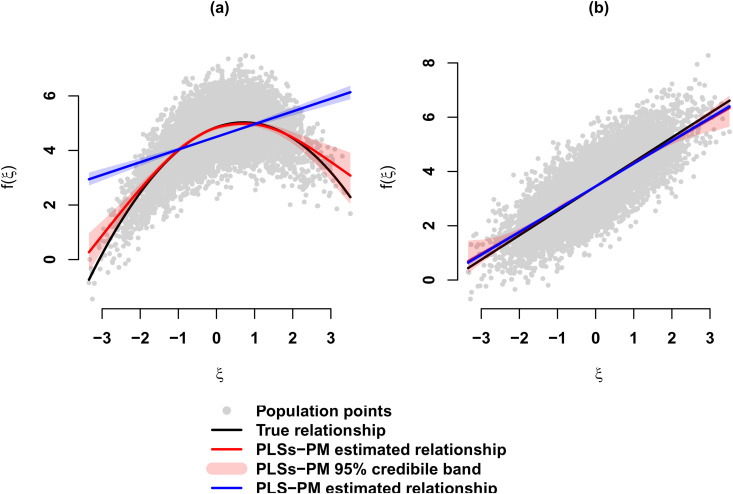
Estimated relationships between *ξ* and $\eta_{\text{4}}$ and $\eta_{\text{5}}$ of the simulated data set of example II. The population factor scores are represented as dots in light gray. The solid black line corresponds to the true relationship. The solid blue line corresponds to the relationship as estimated by PLS-PM algorithm. The solid red line and the light red shaded area corresponds to the relationship as estimated by PLSs-PM algorithm and the corresponding 95% credibility intervals.

For illustration purposes, Fig 11 represents the true (population) relationships and the estimated ones by PLS-PM and PLSs-PM; in the latter case, the 95% credibility bands between *ξ* and $\eta_{1}$, a quadratic relationship, and *ξ* and $\eta_{5}$, the linear relationship. Fig 11(a) illustrates, among other things, the results of S7 Table, in particular, the excellent performance of PLSs-PM in terms of absolute bias and mean square error. Fig 11(b) shows that the results of PLS-PM and PLSs-PM are almost visually identical when the structural relationships are linear, although Table S7 Table demonstrates that, in this case, PLS-PM consistently outperforms the PLSs-PM algorithm.

An analysis of variance (ANOVA) was conducted using the method (PLS-PM vs. PLSs-PM), communality levels (*h* = 25%, 50%, and 75%), and sample sizes (*n* = 75, 100, 150, 250, 300, 500, 750, 900) as factors, along with their interactions, to evaluate effects on absolute bias and RMSE across five structural equations ($\eta_{1}$ to $\eta_{5}$). The detailed results are presented in S8 Table (absolute bias) and S9 Table (RMSE).

Regarding **absolute bias** (S8 Table), the PLSs-PM method significantly reduced bias across most functional forms ($\eta_{1}$ to $\eta_{4}$) compared to standard PLS-PM. However, for the linear functional form $\eta_{5}$, the difference was not statistically significant. The sample size factor alone had limited significance, but its interaction with the method became important – larger sample sizes (particularly n≥150) amplified the benefits of PLSs-PM for bias reduction in non-linear relationships. Communality levels affected results only in the linear case, where bias decreased as communality increased. Interaction terms between method and communality were generally not significant for $\eta_{5}$, reinforcing that nonlinearity primarily benefits from the proposed approach.

For **RMSE** (S9 Table), the PLSs-PM method again showed a statistically significant advantage for most structural relationships, especially non-linear ones. Communality levels were a relevant factor in reducing RMSE for $\eta_{3}$, $\eta_{4}$, and $\eta_{5}$, with higher communality contributing to lower errors. Sample size alone did not yield significant RMSE reductions. However, interaction terms between method and sample size were highly significant for all non-linear functions ($\eta_{1}$, $\eta_{2}$, $\eta_{3}$ and $\eta_{4}$), indicating that the error reduction gains of PLSs-PM are more pronounced with increased sample sizes. Notably, for $\eta_{5}$, RMSE differences were minor, confirming that the added complexity of PLSs-PM is not necessary when the underlying relationship is linear.

These results confirm the advantages of PLSs-PM in detecting and approximating nonlinear relationships, particularly when communality is moderate to high and when sufficient sample size is available.

These results confirm that PLSs-PM consistently produces better levels of absolute bias and mean square error for nonlinear relationships. Indeed, PLSs-PM tends to produce a smaller absolute bias for nonlinear relationships whose functional forms are of the type of equations tested. This is generally true for all levels of communality, although the differences between the two methods increase as communality is moderate to high and when sufficient sample size is available.

## Discussion

This study proposed and tested a new inner weighting scheme and structural relationships estimation method in PLS-PM algorithm. We compared the traditional PLS-PM and the proposed PLSs-PM approaches for the inner approximation of latent variable proxies and structural relationships in the context of Partial Least Squares. A method using additive models based on cubic regression splines was embedded in the PLS-PM algorithm using the Wold’s hybrid approach, *smooth weighting*. The novel inner weighting scheme was introduced and used to estimate two structural equation models using both real and simulated data. Regarding the real data case, although it was not possible to compare numeric measures of accuracy for the real dataset, visual inspection of the estimated structural relationships, as well as the determinantion coefficients, indicated that PLSs-PM can capture nonlinear structural relationships.

The two methods were compared using a simulated dataset that mimicked four nonlinear structural relationships and a linear relationship as case controls. Several scenarios were tested for different levels of communality between the latent variables, observed indicators, and eight different sample sizes. The methods were compared based on the absolute bias and root mean square error. PLSs-PM performed globally better than PLS-PM, except for linear relationships, although the differences between the two were less obvious for very small sample sizes. This result is in line with expectations as the estimation of nonlinear functions demanding in terms of factor determination (i.e., the informativeness of the observed data). Another important observation is the observed ability of PLSs-PM to adjust to linear relationships, as the bias diminishes consistently with the sample size. Nevertheless, for exact linear relationships, the PLSs-PM’s RMSE tends to be larger than that of PLS-PM. This is a result of the increased variance in the estimation of PLSs-PM, as it is more demanding in the number of parameters to estimate.

Although PLSs-PM does not account for PLS-PM inconsistency, it succeeds in it main aim: maximise the explained variance of the endogenous latent variables due to its ability of approximating structural relationships whose functional form is not known and cannot be *a priori* specified as linear. Nevertheless, the fact that the approximation relies on linear combinations of piecewise functions of the predecessor latent variables, it suggests future possible adaptations to produce consistent estimates of structural relationships.

Structural equation models are commonly used in many scientific areas, including social, behavioural, health, and management sciences, to evaluate relationships among latent variables that cannot be observed directly or without noise. Without any empirical or theoretical verification, it is generally assumed that the latent variables are linearly related. As discussed by [25], this is an exception, suggesting that scatter plots between observed variables should be inspected for potential nonlinear trends. Nevertheless, it is admited that the contamination of manifest variables by measurement errors might mask the underlying nonlinear relationships and suggest detection procedures at the level of the structural model.

Extensive attention has been paid to modelling nonlinear trends using quadratic or product-interaction terms, without considering that one rarely has prior knowledge of the nature of the relationships, and that frequently they present patterns that can not be adequately represented by these specific structures. The notable absence of research on methods for estimating nonlinear relationships in structural equation modelling contrasts with the extensive use of (general) additive models for observed (non-latent) variables.

There is little reason to believe that nonlinear effects are less common for latent variables than observed variables. If anything, the nonlinear effects might be harder to visualise and detect with observed variables owing to contamination by measurement error. Therefore, the absence of discussion on techniques for detecting and estimating nonlinear effects in latent variable models is likely related to the fact that few techniques have been proposed or evaluated for this purpose. Because the main consequences of using traditional linear PLS-PM in the presence of structural nonlinear relationships might be a lack of ability to understand the true nature of the relationships between constructs, potential underestimation or overestimation of the relative importance of the drivers of endogenous latent constructs and the fact that estimates of model parameters (loadings, regression coefficients) are biased or suboptimal, it was our goal to propose a new technique and compare its performance with traditional linear PLS-PM.

Future work should be performed to test the performance of these two methods under different conditions, namely when other types of smoothers and/or other types of knot definitions are considered. Improvements on the edges of the relationships are particularly needed, and natural cubic splines are limited to linear relationships beyond the lowest and highest generated proxy values. Moreover, it is important to test whether the performance of the PLSs-PM method is different in the case formative constructs. It is even more important to provide PLSs-PM with automatic mechanisms to detect the presence of nonlinearity and automatically choose a linear or nonlinear estimation procedure. Statistical tests of the additive components of the smoothers can maintain the algorithm on the correct path.

This new methodology may be useful for modelling nonlinear relationships between latent variables and indicators as well as for formative or reflective measurement models.

## Supporting information

S1 Table Measurement model for reduced European Satisfaction Satisfaction Index (ECSI) – banking sector. Latent variables and their indicators: all indicators are measured in a 10-point scale, from 1 to 10, where 1 expresses a very negative opinion and 10 a very positive opinion. The values *α* and *ρ* represent Cronbach’s *α* and Dillon-Goldstein’s *ρ*.
(PDF)

S2 Table Outer model weights. Comparison of results obtained with the ECSI dataset of Example I in plspm, SeminR and authors’ implementation (plsExtpm).
(PDF)

S3 Outer model loadings. Comparison of results obtained with the ECSI dataset of Example I in plspm, SeminR and authors’ implementation (plsExtpm).
(PDF)

S4 Table Inner model: Dillon Goldsteins’s *ρ* and R^2^. Comparison of results obtained with the ECSI dataset of Example I in plspm, SeminR and authors’ implementation (plsExtpm).
(PDF)

S5 Table Inner model: path coefficients. Comparison of results obtained with the ECSI dataset of Example I in plspm, SeminR and authors’ implementation (plsExtpm).
(PDF)

S6 Table Inner model: total effects. Comparison of results obtained with the ECSI dataset of Example I in plspm, SeminR and authors’ implementation (plsExtpm).
(PDF)

S1 Text Derivation of population values of endogenous variables used in factor scores scaling. Supplementary results of Example II.
(PDF)

S7 Table PLSs-PM absolute bias and root mean square error as a index number of PLS-PM absolute bias and root mean square error (base 100). Supplementary results of Example II.
(PDF)

S8 Table ANOVA results. **Dependent variable: absolute bias.** Supplementary results of Example II.
(PDF)

S9 Table ANOVA results. **Dependent variable: RMSE.** Supplementary results of Example II.
(PDF)

S1 Appendix Smooth weighting – a new PLS-PM inner weighting scheme. Appendix [42–55].
(PDF)
