# Supplementary material for: Beyond linearity - a new Partial Least Squares - Path Modelling (PLS-PM) inner weighting scheme for detecting and approximating nonlinear structural relationships in Structural Equation Models
Source: PLoS One. 2026 Mar 23;21(3):e0345111. doi: 10.1371/journal.pone.0345111 (PMC13008259; doi:10.1371/journal.pone.0345111)
Supplement: S1 Table — Latent variables and their indicators: all indicators are measured in a 10-point scale, from 1 to 10, where 1 expresses a very negative opinion and 10 a very positive opinion. The values α and ρ represent Cronbach’s α and Dillon-Goldstein’s ρ. (PDF) [file pone.0345111.s001.pdf]

Table S1: Measurement model for reduced European Satisfaction Satisfaction Index (ECSI) - banking sector. Latent variables and their indicators: all indicators are measured in a 10-point scale, from 1 to 10, where 1 expresses a very negative opinion and 10 a very positive opinion. The values α and ρ represent Cronbach's α and Dillon-Goldstein's ρ.

Reliability	Indicator
**Perceived quality**	
	(a) Overall perceived quality (QUAL1)
	(b) Quality of products and services (QUAL2)
	(c) Customer service and personal advice (QUAL3)
	(d) Availability of contact channels (QUAL4)
	(e) Reliability of products and services (QUAL5)
	(f) Diversity of products and services (QUAL6)
	(g) Clarity and transparency of information provided (QUAL7)
	(h) Accessibility (QUAL8)
	(i) Quality of physical facilities (QUAL9)
**Perceived value**	
$\alpha = 0.9$	(a) Evaluation of price given quality (VALU1)
$\rho = 0.95$	(b) Evaluation of quality given price (VALU2)
**Customer satisfaction**	
$\alpha = 0.84$	(a) Overall satisfaction (SATI1)
$\rho = 0.91$	(b) Fulfilment of expectations (SATI2)
	(c) Distance to ideal bank (SATI3)
**Customer loyalty**	
$\alpha = 0.82$	(a) Intention to remain as a customer (LOYA1)
$\rho = 0.92$	(b) Recommendation to friends and colleagues (LOYA2)
