# Supplementary material for: Beyond linearity - a new Partial Least Squares - Path Modelling (PLS-PM) inner weighting scheme for detecting and approximating nonlinear structural relationships in Structural Equation Models
Source: PLoS One. 2026 Mar 23;21(3):e0345111. doi: 10.1371/journal.pone.0345111 (PMC13008259; doi:10.1371/journal.pone.0345111)
Supplement: S2 Table — Comparison of results obtained with the ECSI dataset of Example I in plspm, SeminR and authors’ implementation (plsExtpm). (PDF) [file pone.0345111.s002.pdf]

Table S2: Outer model weights. Comparison of results obtained with the ECSI dataset of Example I in extitplspm, extitSeminR and authors' implementation.

Indicator	`plspm`	`SeminR`	`plsEXTpm`
**Quality**			
QUAL1	0.40	0.40	0.40
QUAL2	0.24	0.24	0.24
QUAL3	-0.11	-0.11	-0.11
QUAL4	0.10	0.10	0.10
QUAL5	0.01	0.01	0.00
QUAL6	0.40	0.40	0.40
QUAL7	0.06	0.06	0.07
QUAL8	0.11	0.11	0.09
QUAL9	-0.03	-0.03	-0.02
**Value**			
VALU1	0.50	0.50	0.50
VALU2	0.55	0.55	0.55
**Satisfaction**			
SATI1	0.35	0.35	0.35
SATI2	0.40	0.40	0.40
SATI3	0.39	0.39	0.39
**Loyalty**			
LOYA1	0.52	0.52	0.52
LOYA2	0.57	0.57	0.57
