# Supplementary material for: Beyond linearity - a new Partial Least Squares - Path Modelling (PLS-PM) inner weighting scheme for detecting and approximating nonlinear structural relationships in Structural Equation Models
Source: PLoS One. 2026 Mar 23;21(3):e0345111. doi: 10.1371/journal.pone.0345111 (PMC13008259; doi:10.1371/journal.pone.0345111)
Supplement: S3 — Comparison of results obtained with the ECSI dataset of Example I in plspm, SeminR and authors’ implementation (plsExtpm). (PDF) [file pone.0345111.s003.pdf]

Table S3: Outer model loadings. Comparison of results obtained with the ECSI dataset of Example I in extitplspm, extitSeminR and authors' implementation.

Indicator	plspm	SeminR	plsEXTpm
**Value**			
VALU1	0.95	0.95	0.95
VALU2	0.96	0.96	0.96
**Satisfaction**			
SATI1	0.86	0.86	0.86
SATI2	0.88	0.88	0.88
SATI3	0.88	0.88	0.88
**Loyalty**			
LOYA1	0.91	0.91	0.91
LOYA2	0.93	0.93	0.93
