# Supplementary material for: Beyond linearity - a new Partial Least Squares - Path Modelling (PLS-PM) inner weighting scheme for detecting and approximating nonlinear structural relationships in Structural Equation Models
Source: PLoS One. 2026 Mar 23;21(3):e0345111. doi: 10.1371/journal.pone.0345111 (PMC13008259; doi:10.1371/journal.pone.0345111)
Supplement: S4 Table — Comparison of results obtained with the ECSI dataset of Example I in plspm, SeminR and authors’ implementation (plsExtpm). (PDF) [file pone.0345111.s004.pdf]

Table S4: Inner model: Dillon Goldsteins's ρ and R^2

	Value	Satisfaction	Loyalty
**plspm**			
Dillon Goldsteins's ρ	0.95	0.91	0.92
R^2 (%)	37.09	83.42	52.16
**SeminR**			
Dillon Goldsteins's ρ	0.91	0.95	0.92
R^2 (%)	36.95	83.6	52.17
**plsEXTpm**			
Dillon Goldsteins's ρ	0.95	0.91	0.92
R^2 (%)	60.9	91.34	72.22
