# Supplementary material for: Beyond linearity - a new Partial Least Squares - Path Modelling (PLS-PM) inner weighting scheme for detecting and approximating nonlinear structural relationships in Structural Equation Models
Source: PLoS One. 2026 Mar 23;21(3):e0345111. doi: 10.1371/journal.pone.0345111 (PMC13008259; doi:10.1371/journal.pone.0345111)
Supplement: S5 Table — Comparison of results obtained with the ECSI dataset of Example I in plspm, SeminR and authors’ implementation (plsExtpm). (PDF) [file pone.0345111.s005.pdf]

Table S5: Inner model: path coefficients

| | Value | Satisfaction | Loyalty |
|---|---|---|---|
| **plspm** | | | |
| Quality | 0.61 | 0.74 | |
| Value | | 0.25 | |
| Satisfaction | | | 0.72 |
| **SeminR** | | | |
| Quality | 0.61 | 0.74 | |
| Value | | 0.25 | |
| Satisfaction | | | 0.72 |
| **Author's implementation** | | | |
| Quality | 0.61 | 0.74 | |
| Value | | 0.25 | |
| Satisfaction | | | 0.72 |
