# Supplementary material for: Beyond linearity - a new Partial Least Squares - Path Modelling (PLS-PM) inner weighting scheme for detecting and approximating nonlinear structural relationships in Structural Equation Models
Source: PLoS One. 2026 Mar 23;21(3):e0345111. doi: 10.1371/journal.pone.0345111 (PMC13008259; doi:10.1371/journal.pone.0345111)
Supplement: S6 Table — Comparison of results obtained with the ECSI dataset of Example I in plspm, SeminR and authors’ implementation (plsExtpm). (PDF) [file pone.0345111.s006.pdf]

Table S6: Inner model: total effects. Comparison of results obtained with the ECSI dataset of Example I in extitplspm, extitSeminR and authors' implementation.

	Satisfaction	Value	Loyalty
**plspm**			
Quality	0.89	0.61	0.64
Satisfaction			0.72
Value	0.25		0.18
Loyalty			
**SeminR**			
Quality	0.89	0.61	0.64
Satisfaction			0.72
Value	0.25		0.18
Loyalty			
**Author's implementation**			
Quality	0.89	0.61	0.64
Satisfaction			0.72
Value	0.25		0.18
Loyalty			
