# Supplementary material for: Beyond linearity - a new Partial Least Squares - Path Modelling (PLS-PM) inner weighting scheme for detecting and approximating nonlinear structural relationships in Structural Equation Models
Source: PLoS One. 2026 Mar 23;21(3):e0345111. doi: 10.1371/journal.pone.0345111 (PMC13008259; doi:10.1371/journal.pone.0345111)
Supplement: S1 Text — Supplementary results of Example II. (PDF) [file pone.0345111.s007.pdf]

Supplementary results of Example II - Derivation of population values of endogenous variables used in factor scores scaling

The following paragraphs present the calculations to find the population expected values and variances of the five endogenous variables of the structural model of Example III. These population value were used to scale the PLS-PM and PLSs-PM factor scores computed in the last iteration of the algorithms. These scaled scores allow direct comparison with functional relationships described in Figure @ref(fig:fig-ex2-relationships), in @ref(sec:data-example2) and presented in Figure @ref(fig:fig-ex2-results), as well as the computation of Absolute Bias and Root Mean Square Error reported in Figures @ref(fig:fig-ex2-results-bias), @ref(fig:fig-ex2-results-rmse), in @ref(sec:res-example2), and Tables @ref(tab:tab-bias-rmse-index), @ref(tab:tab-bias-anova) and @ref(tab:tab-rmse-anova).

The latent variable η_1 is a function of ξ and ϵ_1, both following standard normal distribution. It is described by $\eta_1 = g(\xi, \epsilon_1) = 4.85 + 0.5\xi - 0.35\xi^2 + \epsilon_1$. We can use the properties of mathematical expectation and variance.

The exogenous random variable follows a standard normal distribution, $\xi \sim N(0,1)$. The perturbances $\epsilon_i \sim N(0, 0.5)$, $i = 1, 2, 3, 4, 5$, and are independent of each other and of ξ.

Hence, the expected value of η_1 is given by:

$$\begin{aligned} \mathrm{E}(\eta_1) &= \mathrm{E}(4.85 + 0.5\xi - 0.35\xi^2 + \epsilon_1) \Leftrightarrow \\ &= \mathrm{E}(4.85) + 0.5\mathrm{E}(\xi) - 0.35\mathrm{E}(\xi^2) + \mathrm{E}(\epsilon_1) \Leftrightarrow \\ &= 4.85 - 0.35 \times 1 + 0 = 4.5. \end{aligned}$$

Assuming that ϵ_1 is independent from ξ, variance of ξ is given by:

$$\begin{aligned} \mathrm{Var}(\eta_1) &= \mathrm{Var}(4.85 + 0.5\xi - 0.35\xi^2 + \epsilon_2) \Leftrightarrow \\ &= \mathrm{Var}(4.85) + 0.5^2\mathrm{Var}(\xi) + 0.35^2\mathrm{Var}(\xi^2) + \mathrm{Var}(\epsilon_1). \end{aligned}$$

As $\mathrm{Var}(\xi) = 1$ and ξ^2 follows a χ^2 distribution with 1 degree of freedom, which implies that $\mathrm{Var}(\xi^2) = 2$, and $\mathrm{Var}(\epsilon_1) = 0.5$;

$$\mathrm{Var}(\eta_1) = 0.25 \cdot 1 + 0.35^2 \cdot 2 + 0.5 = 0.995.$$

The latent variable η_2 is a function of ξ described by $\eta_2 = g(\xi) = 5 - 0.5\xi^2 + \epsilon_2$. Its expected value is given by:

$$\begin{aligned} \mathrm{E}(\eta_2) &= \mathrm{E}(5 - 0.5\xi^2 + \epsilon_{\eta_2}) \Leftrightarrow \\ &= 5 - 0.5\mathrm{E}(\xi^2) + \mathrm{E}(\epsilon_{\eta_2}) \Leftrightarrow \\ &= 5 - 0.5 \times 1 + 0 = 4.5. \end{aligned}$$

Its variance, assuming η_2 is independent of ϵ_{η_2} and taking into account the same properties of ξ^2 mentioned

above, is given by:

$$\begin{aligned}\text{Var}(\eta_2) &= \text{Var}(5 - 0.5\xi^2 + \epsilon_{\eta_2}) \Leftrightarrow \\ &= 0.5^2\text{Var}(\xi^2) + \text{Var}(\epsilon_{\eta_2}) \Leftrightarrow \\ &= 0.25 \cdot 2 + \sigma^2_{\epsilon_{\eta_2}} \Leftrightarrow \\ &= 0.5 + 0.5 = 1.\end{aligned}$$

The latent variable η_3 is a function of ξ described by $\eta_3 = g(\xi) = 5 + 0.04(1 - e^{-1.5\xi}) + \epsilon_{\eta_3}$.

Assuming ϵ_4 is independent of ξ and it determines η_3 additively, we use the change of variable technique to find the distribution of $\eta_3^* = h(\xi) = 5 + 0.04(1 - e^{-1.5\xi})$ in first place.

The transformation of a random variable ξ through a deterministic function is a straightforward application of probability theory. To find the density function of ξ, we can use the probability density function transformation method.

Let $\eta_3^* = h(\xi) = 5 + 0.04(1 - e^{-1.5\xi})$.

The probability density function of η_4 is given by $f_{\eta_3}(\eta_3) = f_\xi(h^{-1}(\eta_3^*))|(h^{-1})'(\eta_3^*)|$, where $(h^{-1})'(\eta_3)$ is the derivative of $h^{-1}(\eta_3^*)$ and f_ξ is the probability density function of the standard normal variable.

Solving $h(\xi)$ for η_3^* we obtain:

$$\begin{aligned}\eta_3^* &= 5 + 0.04(1 - e^{-1.5\xi}) \Leftrightarrow \\ \eta_3^* - 5 &= 0.04(1 - e^{-1.5\xi}) \Leftrightarrow \\ \frac{\eta_3^* - 5}{0.04} &= 1 - e^{-1.5\xi} \Leftrightarrow \\ 1 - \frac{\eta_3^* - 5}{0.04} &= e^{-1.5\xi} \Leftrightarrow \\ \log\left(1 - \frac{\eta_3^* - 5}{0.04}\right) &= -1.5\xi \Leftrightarrow \\ \xi &= -\frac{1}{1.5}\log\left(\frac{0.04 - \eta_3^* + 5}{0.04}\right).\end{aligned}$$

The derivative of $(h^{-1})'(\eta_3^*)$ is:

$$\begin{aligned}(h^{-1})'(\eta_3^*) &= -\frac{1}{1.5}\log\left(\frac{0.04 - \eta_3^* + 5}{0.04}\right) \Leftrightarrow \\ &= -\frac{1}{1.5}\left[\log\left(\frac{0.04 - \eta_3^* + 5}{0.04}\right)\right]' \Leftrightarrow \\ &= -\frac{1}{1.5}\frac{1}{\left(\frac{0.04 - \eta_3^* - 5}{0.04}\right)}\left(\frac{0.04 - \eta_3^* + 5}{0.04}\right)' \Leftrightarrow \\ &= -\frac{1}{1.5}\frac{1}{\frac{0.04 - \eta_3^* + 5}{0.04}}\left(-\frac{1}{0.04}\right) \Leftrightarrow \\ &= \frac{25 \cdot 0.04}{1.5(0.04 - \eta_3^* + 5)} \Leftrightarrow \\ &= \frac{1}{1.5(0.04 - \eta_3^* + 5)}.\end{aligned}$$

Now, substitute $h^{-1}(\eta_3^*)$ and $(h^{-1})'(\eta_3^*)$ into the density function formula

$$f_{\eta_3^*}(\eta_3^*) = f_\xi\left(-\frac{1}{1.5}\log\left(\frac{0.04-\eta_3^*+5}{0.04}\right)\right)\left|\frac{1}{1.5(0.04-\eta_3^*+5)}\right| \Leftrightarrow$$
$$= \frac{1}{\sqrt{2\pi}1.5(0.04-\eta_3^*+5)} e^{-\frac{1}{2}\left(\frac{1}{1.5}\log\left(\frac{0.04-\eta_3^*+5}{0.04}\right)\right)^2}.$$

The support of η_3^* is given by

$$\begin{aligned}
&-\infty \leq \xi \leq +\infty \Leftrightarrow\\
&-\infty \leq -1.5\xi \leq +\infty \Leftrightarrow\\
&-\infty \leq -e^{-1.5\xi} \leq 0 \Leftrightarrow\\
&-\infty \leq 1-e^{-1.5\xi} \leq 1 \Leftrightarrow\\
&-\infty \leq 0.04(1-e^{-1.5\xi}) \leq 0.04 \Leftrightarrow\\
&-\infty \leq 5+0.04(1-e^{-1.5\xi}) \leq 5.04. -\infty \qquad \leq \eta_3^* \leq 5.04.
\end{aligned}$$

Therefore the expected value of η_3^* is given by

$$\begin{aligned}
\mathrm{E}(\eta_3^*) &= \int_{-\infty}^{5.04} \eta_3^* \cdot \frac{1}{\sqrt{2\pi}1.5(0.04-\eta_3^*-5)} e^{-\frac{1}{2}\left(\frac{1}{1.5}\log\left(\frac{0.04-\eta_3^*+5}{0.04}\right)\right)^2} d\eta_3^* \Leftrightarrow\\
&= 4.9168.
\end{aligned}$$

Consequently, the expected value of η_3 is:

$$\mathrm{E}(\eta_3) = \mathrm{E}(\eta_3^*) + \mathrm{E}(\epsilon_3) = 4.9168.$$

The variance of η_3^* can be obtained through the equation $\mathrm{Var}(\eta_3^*) = \mathrm{E}((\eta_3^*)^2) - \mathrm{E}(\eta_3^*)^2$. The first term of the right-hand side of theis equation is given by:

$$\begin{aligned}
\mathrm{E}(\eta_3^2) &= \int_{-\infty}^{5.04} \eta_3^2 \cdot \frac{1}{\sqrt{2\pi}1.5(0.04-\eta_3-5)} e^{-\frac{1}{2}\left(\frac{1}{1.5}\log\left(\frac{0.04-\eta_3+5}{0.04}\right)\right)^2} d\eta_3 \Leftrightarrow\\
&= 24.3037.
\end{aligned}$$

Consequently, the variance of η_3^* is:

$$\begin{aligned}
\mathrm{Var}(\eta_3^*) &= \mathrm{E}((\eta_3^*)^2) - \mathrm{E}(\eta_3^*)^2 \Leftrightarrow\\
&= 0.1288.
\end{aligned}$$

Finally, the variance of η_3 is give by:

$$\begin{aligned}
\mathrm{Var}(\eta_3) &= \mathrm{Var}(\eta_3^*) + \mathrm{Var}(\epsilon\eta_3) \Leftrightarrow\\
&= 0.1288 + 0.5 = 0.6288.
\end{aligned}$$

The latent variable η_4 is a function of ξ described by the following expression:

$$\eta_4 = g(\xi) = \begin{cases} 5+0.1\xi-0.3\xi^2+\epsilon_4, & \xi < 0\\ 5+0.1\xi-0.1\xi^2+\epsilon_4, & \xi > 0. \end{cases}$$

As $\xi \sim N(0,1)$, $\mathrm{P}(\xi < 0) = \mathrm{P}(\xi > 0) = 0.5$, thus

$$\eta_4 = g(\xi) = \begin{cases} 5+0.1\xi-0.3\xi^2+\epsilon_4, & \text{with probability } p = 0.5\\ 5+0.1\xi-0.1\xi^2+\epsilon_4, & \text{with probability } p = 0.5. \end{cases}$$

Its expected value is given by:

$$\begin{aligned}
\mathrm{E}(\eta_4) &= 0.5\left(\mathrm{E}(5+0.1\xi-0.3\xi^2+\epsilon_4\right)+0.5\left(\mathrm{E}(5+0.1\xi-0.1\xi^2\epsilon_{\eta_2}+\epsilon_4)\right) \Leftrightarrow \\
&= 0.5\left(5+0.1\mathrm{E}(\xi)-0.3\mathrm{E}(\xi^2)+\mathrm{E}(\epsilon_4^2)\right)+0.5\left(5+0.1\mathrm{E}(\xi)-0.1\mathrm{E}(\xi^2)+\mathrm{E}(\epsilon_4^2)\right) \Leftrightarrow \\
&= 0.5(5-0.3\cdot 1)+0.5(5-0.1\cdot 1)=4.5
\end{aligned}$$

Its variance, assuming η_4 is independent of ϵ_{η_4} by:

$$\begin{aligned}
\mathrm{Var}(\eta_4) &= 0.5\left(\mathrm{Var}(5+0.1\xi-0.3\xi^2+\epsilon_4\right)+0.5\left(\mathrm{Var}(5+0.1\xi-0.1\xi^2\epsilon_{\eta_2}+\epsilon_4)\right) \Leftrightarrow \\
&= 0.5\left(0.1^2\mathrm{Var}(\xi)+0.3^2\mathrm{Var}(\xi^2)+\mathrm{Var}(\epsilon_4^2)\right)+0.5\left(0.1^2\mathrm{Var}(\xi)+0.1^2\mathrm{Var}(\xi^2)+\mathrm{Var}(\epsilon_4^2)\right) \Leftrightarrow \\
&= 0.5(0.1^2\cdot 1+0.3^2\cdot 2+0.5)+0.5(0.1^2\cdot 1+0.1^2\cdot 2+0.5)=0.61
\end{aligned}$$

The latent variable η_5 is a function of ξ described by $\eta_5 = g(\xi) = 3.45+0.9\xi+\epsilon_5$. It is a linear transformation of ξ, therefore its expected value is given by:

$$\begin{aligned}
\mathrm{E}(\eta_5) &= \mathrm{E}(3.45+0.9\xi+\epsilon_5) \Leftrightarrow \\
&= \mathrm{E}(3.45)+0.9\mathrm{E}(\xi)+\mathrm{E}(\epsilon_5) \Leftrightarrow \\
&= 3.45.
\end{aligned}$$

Its variance, assuming ξ is independent of ϵ_5, is given by:

$$\begin{aligned}
\mathrm{Var}(\eta_5) &= \mathrm{Var}(3.45+0.9\xi+\epsilon_5) \Leftrightarrow \\
&= 0.9^2\mathrm{Var}(\xi)+\mathrm{Var}(\epsilon_5) \Leftrightarrow \\
&= 0.81+\sigma^2_{\epsilon_5} \Leftrightarrow \\
&= 0.81+0.5=1.31.
\end{aligned}$$
