# Supplementary material for: Beyond linearity - a new Partial Least Squares - Path Modelling (PLS-PM) inner weighting scheme for detecting and approximating nonlinear structural relationships in Structural Equation Models
Source: PLoS One. 2026 Mar 23;21(3):e0345111. doi: 10.1371/journal.pone.0345111 (PMC13008259; doi:10.1371/journal.pone.0345111)
Supplement: S7 Table — Supplementary results of Example II. (PDF) [file pone.0345111.s008.pdf]

Table S7: PLSs-PM absolute bias and root mean square error as a index number of PLS-PM absolute bias and root mean square error (base 100). Supplementary results of Example II

	Bias					RMSE				
Sample size	η_1	η_2	η_3	η_4	η_5	η_1	η_2	η_3	η_4	η_5
**Communality = 25%**										
n100	65.00	59.08	75.52	70.72	99.85	76.45	66.79	99.59	90.48	119.33
n150	61.90	57.64	73.39	75.45	100.63	69.88	63.17	95.09	88.95	110.62
n250	61.00	57.27	73.77	72.47	101.00	66.38	61.74	90.10	82.22	106.80
n300	60.91	57.54	72.16	70.45	101.93	65.44	61.63	87.93	79.37	106.86
n500	58.72	56.78	69.89	68.45	102.18	62.06	59.90	84.15	75.29	105.52
n75	63.61	58.29	73.94	74.30	96.54	78.75	68.30	103.12	97.16	122.79
n750	57.91	55.77	68.27	67.88	102.32	60.53	58.66	81.73	73.37	105.07
n900	58.56	56.35	70.19	68.41	102.43	61.06	59.05	82.12	73.77	105.02
**Communality = 50%**										
n100	39.57	37.46	58.18	50.66	108.12	53.10	48.06	89.21	72.20	154.22
n150	37.40	35.78	55.73	48.94	105.93	48.01	44.31	83.51	66.08	142.03
n250	35.66	33.95	53.77	44.30	104.10	43.16	40.68	77.27	58.13	125.92
n300	34.26	32.91	51.66	44.30	105.59	41.26	39.09	74.24	57.02	125.69
n500	33.04	31.37	51.65	41.80	104.82	38.37	36.03	69.87	52.14	118.31
n75	40.44	38.02	62.74	50.80	106.17	57.48	52.24	94.70	78.21	168.92
n750	32.32	30.57	49.84	39.35	105.17	37.15	34.54	65.74	48.64	114.74
n900	31.98	30.56	49.89	38.99	106.26	36.41	34.19	63.78	47.84	115.72
**Communality = 75%**										
n100	24.01	22.66	49.88	38.42	98.93	41.00	35.62	81.45	60.69	216.65
n150	22.18	21.25	44.80	36.28	108.37	35.85	31.88	74.32	53.61	201.36
n250	19.13	18.63	37.50	30.85	106.97	29.56	27.36	65.07	44.88	175.36
n300	18.69	18.10	38.89	30.89	110.69	28.44	26.16	64.46	43.42	174.39
n500	16.86	16.41	33.95	29.40	107.42	24.25	23.08	56.69	38.61	145.39
n75	26.04	24.75	53.85	39.52	111.03	45.73	39.30	86.93	68.12	255.00
n750	15.55	15.57	30.52	26.82	108.42	21.88	21.28	51.10	34.67	137.24
n900	15.43	15.28	29.55	26.76	106.98	21.34	20.74	48.47	33.72	131.77
