# Supplementary material for: Beyond linearity - a new Partial Least Squares - Path Modelling (PLS-PM) inner weighting scheme for detecting and approximating nonlinear structural relationships in Structural Equation Models
Source: PLoS One. 2026 Mar 23;21(3):e0345111. doi: 10.1371/journal.pone.0345111 (PMC13008259; doi:10.1371/journal.pone.0345111)
Supplement: S8 Table — Dependent variable: absolute bias. Supplementary results of Example II. (PDF) [file pone.0345111.s009.pdf]

Table S8: ANOVA results. Dependent variable: absolute bias. p-values are indicated within brackets next to parameter estimates. Supplementary results of Example II.

Factor | Level	η_1	η_2	η_3	η_4	η_5
**Sample size**					
$n = 100$	0.000 (0.997)	-0.001 (0.970)	-0.003 (0.771)	-0.001 (0.907)	0.001 (0.897)
$n = 150$	0.000 (0.968)	-0.001 (0.958)	-0.006 (0.616)	-0.002 (0.832)	0.005 (0.600)
$n = 250$	0.000 (0.953)	0.000 (0.970)	-0.007 (0.528)	-0.002 (0.831)	0.008 (0.409)
$n = 300$	0.000 (0.940)	0.000 (0.975)	-0.008 (0.456)	-0.002 (0.797)	0.008 (0.389)
$n = 500$	0.000 (0.996)	0.000 (0.997)	0.001 (0.913)	0.001 (0.913)	-0.007 (0.489)
$n = 750$	0.001 (0.938)	-0.001 (0.964)	-0.009 (0.429)	-0.002 (0.783)	0.009 (0.341)
$n = 900$	0.001 (0.926)	-0.001 (0.963)	-0.009 (0.400)	-0.002 (0.791)	0.011 (0.278)
**Method**					
PLSs-PM	-0.408 (0.000)	-0.662 (0.000)	-0.118 (0.000)	-0.163 (0.000)	0.002 (0.847)
**Communality**					
h=50%	-0.002 (0.623)	-0.001 (0.951)	0.007 (0.334)	0.003 (0.454)	-0.285 (0.000)
h=75%	-0.001 (0.898)	0.000 (0.960)	0.014 (0.054)	0.006 (0.147)	-0.435 (0.000)
**Method | Communality**					
PLSs-PM|h=50%	-0.295 (0.000)	-0.392 (0.000)	-0.098 (0.000)	-0.170 (0.000)	0.010 (0.251)
PLSs-PM|h=75%	-0.480 (0.000)	-0.639 (0.000)	-0.178 (0.000)	-0.254 (0.000)	0.003 (0.750)
**Method | Sample size**					
PLSs-PM|$n = 100$	-0.028 (0.005)	-0.025 (0.197)	-0.016 (0.311)	0.002 (0.828)	0.003 (0.843)
PLSs-PM|$n = 150$	-0.050 (0.000)	-0.052 (0.011)	-0.031 (0.056)	-0.026 (0.015)	0.001 (0.918)
PLSs-PM|$n = 250$	-0.057 (0.000)	-0.059 (0.004)	-0.035 (0.035)	-0.030 (0.006)	0.006 (0.678)
PLSs-PM|$n = 300$	-0.078 (0.000)	-0.081 (0.000)	-0.047 (0.005)	-0.043 (0.000)	0.004 (0.747)
PLSs-PM|$n = 500$	0.006 (0.529)	0.010 (0.591)	0.012 (0.448)	0.010 (0.323)	-0.003 (0.809)
PLSs-PM|$n = 750$	-0.089 (0.000)	-0.096 (0.000)	-0.059 (0.001)	-0.055 (0.000)	0.005 (0.696)
PLSs-PM|$n = 900$	-0.088 (0.000)	-0.094 (0.000)	-0.057 (0.001)	-0.055 (0.000)	0.006 (0.659)
