# Supplementary material for: Beyond linearity - a new Partial Least Squares - Path Modelling (PLS-PM) inner weighting scheme for detecting and approximating nonlinear structural relationships in Structural Equation Models
Source: PLoS One. 2026 Mar 23;21(3):e0345111. doi: 10.1371/journal.pone.0345111 (PMC13008259; doi:10.1371/journal.pone.0345111)
Supplement: S9 Table — Dependent variable: RMSE. Supplementary results of Example II. (PDF) [file pone.0345111.s010.pdf]

Table S9: ANOVA results. Dependent variable: RMSE. p-values are indicated within brackets next to parameter estimates. Supplementary results of Example II.

Factor | Level	η_1	η_2	η_3	η_4	η_5
**Sample size**					
$n = 100$	-0.003 (0.769)	-0.013 (0.574)	0.000 (0.995)	-0.003 (0.762)	-0.007 (0.731)
$n = 150$	-0.005 (0.632)	-0.024 (0.318)	0.001 (0.950)	-0.002 (0.827)	-0.011 (0.584)
$n = 250$	-0.006 (0.612)	-0.026 (0.281)	0.005 (0.802)	-0.003 (0.781)	-0.009 (0.632)
$n = 300$	-0.007 (0.537)	-0.030 (0.204)	0.008 (0.696)	-0.001 (0.926)	-0.013 (0.512)
$n = 500$	0.003 (0.761)	0.010 (0.673)	0.011 (0.586)	0.006 (0.562)	0.001 (0.953)
$n = 750$	-0.008 (0.500)	-0.033 (0.166)	0.009 (0.648)	-0.001 (0.934)	-0.014 (0.480)
$n = 900$	-0.008 (0.500)	-0.034 (0.156)	0.011 (0.575)	-0.001 (0.961)	-0.013 (0.513)
**Method**					
PLSs-PM	-0.380 (0.000)	-0.739 (0.000)	0.028 (0.215)	-0.064 (0.000)	0.130 (0.000)
**Communality**					
h=50%	-0.011 (0.131)	-0.005 (0.714)	-0.025 (0.043)	-0.027 (0.000)	-0.326 (0.000)
h=75%	-0.007 (0.297)	-0.007 (0.618)	-0.045 (0.001)	-0.037 (0.000)	-0.492 (0.000)
**Method | Communality**					
PLSs-PM|h=50%	-0.370 (0.000)	-0.491 (0.000)	-0.149 (0.000)	-0.206 (0.000)	0.038 (0.032)
PLSs-PM|h=75%	-0.588 (0.000)	-0.791 (0.000)	-0.270 (0.000)	-0.321 (0.000)	0.053 (0.004)
**Method | Sample size**					
PLSs-PM|$n = 100$	-0.089 (0.000)	-0.080 (0.022)	-0.066 (0.024)	-0.045 (0.005)	-0.046 (0.108)
PLSs-PM|$n = 150$	-0.167 (0.000)	-0.148 (0.000)	-0.144 (0.000)	-0.119 (0.000)	-0.087 (0.004)
PLSs-PM|$n = 250$	-0.188 (0.000)	-0.170 (0.000)	-0.166 (0.000)	-0.135 (0.000)	-0.088 (0.004)
PLSs-PM|$n = 300$	-0.243 (0.000)	-0.228 (0.000)	-0.227 (0.000)	-0.179 (0.000)	-0.112 (0.000)
PLSs-PM|$n = 500$	0.060 (0.001)	0.068 (0.048)	0.055 (0.056)	0.062 (0.000)	0.046 (0.106)
PLSs-PM|$n = 750$	-0.270 (0.000)	-0.261 (0.000)	-0.274 (0.000)	-0.208 (0.000)	-0.120 (0.000)
PLSs-PM|$n = 900$	-0.274 (0.000)	-0.264 (0.000)	-0.290 (0.000)	-0.212 (0.000)	-0.121 (0.000)
