# Supplementary material for: Beyond linearity - a new Partial Least Squares - Path Modelling (PLS-PM) inner weighting scheme for detecting and approximating nonlinear structural relationships in Structural Equation Models
Source: PLoS One. 2026 Mar 23;21(3):e0345111. doi: 10.1371/journal.pone.0345111 (PMC13008259; doi:10.1371/journal.pone.0345111)
Supplement: S1 Appendix — Appendix [42–55]. (PDF) [file pone.0345111.s011.pdf]

S1 Appendix. Smooth weighting - a new PLS-PM inner weighting scheme

The PLS-PM algorithm was originally developed by [1] in 1966 and later extended by [2]. This algorithm estimates the path coefficients of the structural model, and the weights and loadings of the manifest variables to maximise the explained variance of the endogenous latent variables. [3] and [4] provide detailed descriptions of the stages of the PLS-PM algorithm. For illustration, we describe the algorithm as presented by [5] and [6] and rely also on [7]. Although this description is well-known, we reproduce it to establish a basis for the presentation of the novel *smooth weighting* scheme. The transcribed text is in italics, except for parts added by the authors.

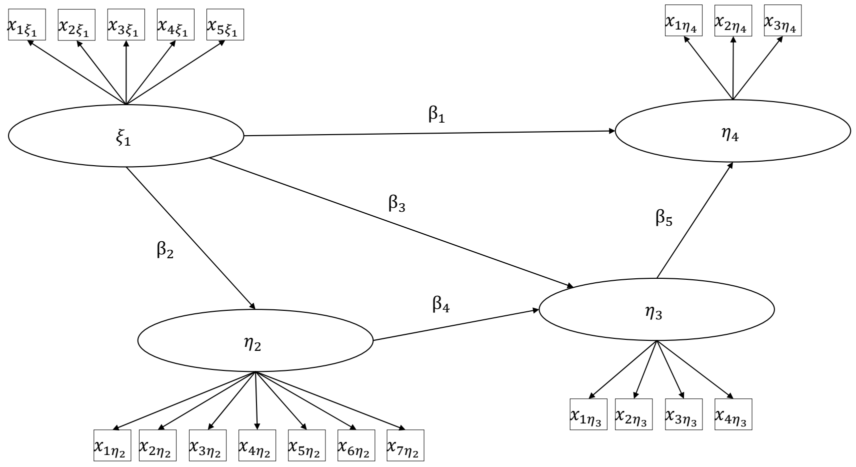

Figure 1: Example of a simple structural equation model with four latent variables and nineteen indicators

The PLS-PM algorithm is essentially a sequence of regressions in terms of weight vectors. [...] The basic PLS-PM algorithm, as suggested by [2], includes the following three stages:

Stage 1: Iterative estimation of latent variables scores, consisting of a four-step iterative procedure that is repeated until convergence is obtained: (1) outer approximation of the latent variable scores, (2) estimation of the inner weights, (3) inner approximation of the latent variable scores,and (4) estimation of the outer weights.

Stage 2: Estimation of outer weights/loadings and path coefficients.

Stage 3: Estimation of location parameters.

[...]

These four steps are repeated until the change in outer weights between two iterations drops below a predefined limit. The algorithm terminates after step 1, delivering latent variable scores for all latent variables. Loadings and inner regression coefficients are then calculated in a straight forward way, given the constructed indices. In order to determine the path coefficients, for each endogenous latent variable a (multiple) linear regression is conducted.

Foreseeing its utility to illustrate our proposed novel approach, the notation used to depict the algorithm steps is established here. The model depicted in Fig 1 is used as a role model.

The structural model depicted in Fig 1 corresponds to the following set of structural equations:

$$\begin{aligned}
\xi_1 &= \xi_1 + 0 \\
\eta_2 &= \beta_2 \xi_1 + \epsilon_1 \\
\eta_3 &= \beta_3 \xi_1 + \beta_4 \eta_2 + \epsilon_2 \\
\eta_4 &= \beta_1 \xi_1 + \beta_5 \eta_3 + \epsilon_3
\end{aligned} \tag{1}$$

We distinguish between exogenous latent variables, not explained by the model and therefore having no arrows ending on them (having no *predecessors*), and represented by ($\xi_{(\bullet)}$), and endogenous latent variables, explained by the model and having arrows ending or leaving them (can have *predecessors* or *successors*), and represented by ($\eta_{(\bullet)}$).

A model adjacency matrix, $\mathbf{D}$ is defined to represent the structural model. The entries of $\mathbf{D}$ are either 0 or 1. If entry $d_{ij} = 1$, latent variable i is a predecessor of latent variable j, and 0 otherwise. The matrix $\mathbf{D}$ is structured as an upper triangular matrix. *Exogenous* latent variables do not have *predecessors* in the structural model and correspond to variables where the *diagonal element is not zero.*

The structural model in Fig 1, for example, has only one exogenous latent variable, (ξ_1). The remaining variables, η_2, η_3, and η_4 are endogenous.

The structural model adjacency matrix $\mathbf{D}$ representing the model of Fig 1 is:

$$\mathbf{D} = \begin{matrix} \\ \xi_1 \\ \eta_2 \\ \eta_3 \\ \eta_4 \end{matrix} \begin{matrix} \begin{matrix} \xi_1 & \eta_2 & \eta_3 & \eta_4 \end{matrix} \\ \begin{pmatrix} 1 & 1 & 1 & 1 \\ 0 & 0 & 1 & 0 \\ 0 & 0 & 0 & 1 \\ 0 & 0 & 0 & 0 \end{pmatrix} \end{matrix} \tag{2}$$

Let us generically denote by $\boldsymbol{\mathcal{V}}' = (\boldsymbol{\xi}', \boldsymbol{\eta}')$ the vector of model's exogenous and endogenous latent variables. Its cardinality, denoted by $\#(\boldsymbol{\mathcal{V}})$ is H, and $\mathcal{V}_h$, $h = 1, \ldots, H$ is the hth element of $\boldsymbol{\mathcal{V}}$. Denote by $\boldsymbol{\beta}$ the matrix containing the structural coefficients, $\beta_{(\bullet)}$, wherever the adjacency matrix $\mathbf{D}'$ has ones, and ones wherever the adjacency matrix $\mathbf{D}'$ has zeros (for exogenous latent variables), and zero elsewhere.

Regarding the model in Fig 1, $\boldsymbol{\mathcal{V}}' = (\xi, \eta_1, \eta_2, \eta_3)$, $H = \#(\boldsymbol{\mathcal{V}}) = 19$ and $\boldsymbol{\beta}$ is:

$$\boldsymbol{\beta} = \begin{matrix} \\ \xi_1 \\ \eta_2 \\ \eta_3 \\ \eta_4 \end{matrix} \begin{matrix} \begin{matrix} \xi_1 & \eta_2 & \eta_3 & \eta_4 \end{matrix} \\ \begin{pmatrix} 1 & 0 & 0 & 0 \\ \beta_2 & 0 & 0 & 0 \\ \beta_3 & \beta_4 & 0 & 0 \\ \beta_1 & 0 & \beta_5 & 0 \end{pmatrix} \end{matrix} \tag{3}$$

Furthermore, let us denote by $\boldsymbol{\varepsilon}'$ the vector of measurement errors of structural equations.

In the case of the model represented in Fig 1, described by the system of Eq (1), $\boldsymbol{\varepsilon}' = (0, \epsilon_2, \epsilon_3, \epsilon_4)$, and the structural model equations can be defined in matrix format as:

$$\boldsymbol{\mathcal{V}} = \boldsymbol{\mathcal{V}}\boldsymbol{\beta} + \boldsymbol{\varepsilon}, \tag{4}$$

where $\boldsymbol{\varepsilon}$ are assumed to be centred, i.e., $\mathrm{E}(\boldsymbol{\varepsilon}) = \mathbf{0}$.

Any exogenous latent variable, such as $\xi_{(\bullet)}$, and any endogenous latent variable, $\eta_{(\bullet)}$ are unobservable variables (or constructs) indirectly described by a block of *manifest variables* $\mathbf{x}_{(\bullet)}$, frequently also called *indicators*.

There are three ways to relate the manifest variables to their latent variables, respectively, called the **reflective** way, the **formative** one, and the **MIMIC** way. The changes in the PLS-PM method we propose in this work concern only the inner model approximation of the latent variable scores and the final estimation of structural relationships. Therefore, we do not detail here none of them as the reader can find a very detailed description in [3].

Let x_{ph}, $p = 1, \ldots, P_h$ and $h = 1, \ldots, H$, be a set of P_h manifest variables or indicators related to latent variable $\mathcal{V}_h$, whatever the way (reflective, formative or MIMIC).

Additionally, let $\mathbf{w}_h$, $h = 1, \ldots, H$, be a column vector of length P_h. The $((P_1 + P_2 + \cdots + P_H) \times H)$ matrix of the outer weights $\mathbf{W}$, referred to in Stage 1 of the PLS-PM algorithm is composed of H blocks, corresponding the indicator vectors $\mathbf{x}'_h = (x_{1h}, \ldots, x_{ph})$, $p = 1, \ldots, P_h$ and $h = 1, \ldots, H$:

$$\mathbf{W} = \begin{matrix} \\ \xi_1 \\ \cdots \\ \xi_h \\ \eta_{h+1} \\ \vdots \\ \eta_H \end{matrix} \begin{matrix} \xi_1 & \cdots & \xi_h & \eta_{h+1} & \cdots & \eta_H \\ \end{matrix} \begin{pmatrix} \mathbf{w}_1 & \cdots & \mathbf{0} & \mathbf{0} & \cdots & \mathbf{0} \\ \mathbf{0} & \ddots & \ddots & \ddots & \ddots & \mathbf{0} \\ \mathbf{0} & \cdots & \mathbf{w}_h & \mathbf{0} & \cdots & \mathbf{0} \\ \mathbf{0} & \cdots & \mathbf{0} & \mathbf{w}_{h+1} & \cdots & \vdots \\ \vdots & \ddots & \ddots & \ddots & \ddots & \mathbf{0} \\ \mathbf{0} & \cdots & \mathbf{0} & \mathbf{0} & \cdots & \mathbf{w}_H \end{pmatrix} \tag{5}$$

Regarding model depicted in Fig 1, $\mathbf{W}$ is:

$$\mathbf{W} = \begin{matrix} \xi_1 \\ \eta_2 \\ \eta_3 \\ \eta_4 \end{matrix} \begin{matrix} \xi_1 & \eta_2 & \eta_3 & \eta_4 \end{matrix} \begin{pmatrix} w_{11} & 0 & 0 & 0 \\ w_{21} & 0 & 0 & 0 \\ w_{31} & 0 & 0 & 0 \\ w_{41} & 0 & 0 & 0 \\ w_{51} & 0 & 0 & 0 \\ 0 & w_{12} & 0 & 0 \\ 0 & w_{22} & 0 & 0 \\ 0 & w_{32} & 0 & 0 \\ 0 & w_{42} & 0 & 0 \\ 0 & w_{52} & 0 & 0 \\ 0 & w_{62} & 0 & 0 \\ 0 & w_{72} & 0 & 0 \\ 0 & 0 & w_{13} & 0 \\ 0 & 0 & w_{23} & 0 \\ 0 & 0 & w_{33} & 0 \\ 0 & 0 & w_{43} & 0 \\ 0 & 0 & 0 & w_{14} \\ 0 & 0 & 0 & w_{24} \\ 0 & 0 & 0 & w_{34} \end{pmatrix} \tag{6}$$

The adjacency matrix of the measurement model, $\mathbf{M}$, has the same structure as the matrix of outer weights $\mathbf{W}$ and it is used for the initialisation, as we will see, when the PLS-PM algorithm is described further ahead. If the entry $m_{ph} = 1$, the manifest variable x_{ph} is one of indicators of latent variable $\mathcal{V}_h$, $p = 1, \ldots, P_h$, $h = 1, \ldots, H$. The matrix $\mathbf{M}$ includes no information about the direction. So it does not tell us anything about the measurement mode of the blocks.

In the model represented in Fig 1, for instance, the manifest variables $x_{1_\xi}, x_{2_\xi}, x_{3_\xi}, x_{4_\xi}, x_{5_\xi}$, are indicators of the latent variable ξ_1. Therefore, the adjacency matrix of the measurement model represented in Fig 1 is:

$$\mathbf{M} = \begin{array}{c} \\ x_{1_{\xi_1}} \\ x_{2_{\xi_1}} \\ x_{3_{\xi_1}} \\ x_{4_{\xi_1}} \\ x_{5_{\xi_1}} \\ x_{1_{\eta_2}} \\ x_{2_{\eta_2}} \\ x_{3_{\eta_2}} \\ x_{4_{\eta_2}} \\ x_{5_{\eta_2}} \\ x_{6_{\eta_2}} \\ x_{7_{\eta_2}} \\ x_{1_{\eta_3}} \\ x_{2_{\eta_3}} \\ x_{3_{\eta_3}} \\ x_{4_{\eta_3}} \\ x_{1_{\eta_4}} \\ x_{2_{\eta_4}} \\ x_{3_{\eta_4}} \end{array} \begin{array}{c} \begin{array}{cccc} \xi_1 & \eta_2 & \eta_3 & \eta_4 \end{array} \\ \begin{pmatrix} 1 & 0 & 0 & 0 \\ 1 & 0 & 0 & 0 \\ 1 & 0 & 0 & 0 \\ 1 & 0 & 0 & 0 \\ 1 & 0 & 0 & 0 \\ 0 & 1 & 0 & 0 \\ 0 & 1 & 0 & 0 \\ 0 & 1 & 0 & 0 \\ 0 & 1 & 0 & 0 \\ 0 & 1 & 0 & 0 \\ 0 & 1 & 0 & 0 \\ 0 & 1 & 0 & 0 \\ 0 & 0 & 1 & 0 \\ 0 & 0 & 1 & 0 \\ 0 & 0 & 1 & 0 \\ 0 & 0 & 1 & 0 \\ 0 & 0 & 0 & 1 \\ 0 & 0 & 0 & 1 \\ 0 & 0 & 0 & 1 \end{pmatrix} \end{array} \tag{7}$$

On the other hand, let $\mathbf{X}$ be the manifest variables matrix, having n rows, the sample size, and $(P_1 + \cdots + P_H)$ columns, the number of manifest variables.

The description of the PLS-PM algorithm and the incorporation of the nonlinearities estimation in inner approximation of the structural model results in a new inner weighting scheme named *smooth weighting* follows. Indeed, as Wold observed, PLS-PM can incorporate nonlinearities in the structural model through the hybrid approach [8] by including internal proxies for each nonlinear term during the iterative PLS-PM algorithm runtime. Based on this concept, we propose a **smoothing-based hybrid approach**.

Splines belong to to the broad class of regression models that are used to estimate a smooth function that represents the underlying trend of a set of data points. They are particularly useful when dealing with noisy data or when the relationship between variables is not well defined. Splines strike a balance between fitting the data closely and ensuring that the estimated curve remains smooth, making them a valuable tool in various scientific disciplines including statistics [9], economics [10], biology [11,12], and engineering [13,14] projects. Smoothing splines can be used as a flexible tool for nonlinear regression, in which the relationship between the response and predictor variables is not linear. They are particularly useful when the functional form of the relationship is unknown.

Regression splines are constructed by selecting a basis and specifying the set of functions for which the function b is an element. This involves selecting basis functions, which are treated as known quantities; for example, if $b_j(x)$ is the jth basis function, then f is represented as follows:

$$f(x; \boldsymbol{\alpha}, \mathbf{b}) = \sum_{j=1}^{K} \alpha_j b_j(x) \tag{8}$$

for some values of the unknown parameters, α_j, $j = 1, \ldots, K$. The model in Eq (8) is linear in the parameters and penalised least squares may be employed to estimate the parameters α_j, $j = 1, \ldots, K$ with an appropriate degree of smoothing.

Here the choice of basis functions relies on the cubic regression splines [15]. They are penalised by the conventional integrated square second derivative cubic spline penalty [15]. Cubic regression splines are a very

flexible class of smoothing functions and they might be combined additively when the dependent variable, y, is a function of several regressors. They result in a piecewise continuous linear additive model as follows:

$$y_i = f_1(x_{1i}; \boldsymbol{\alpha}_1, \mathbf{b}_1, K_1) + \cdots + f_L(x_{Li}; \boldsymbol{\alpha}_L, \mathbf{b}_L, K_L) + \epsilon_i \tag{9}$$

where $x_1, \ldots, x_L$, are regressor variables, f_l, $l = 1, \ldots, L$, are univariate cubic regression splines [15], $\mathbf{b}'_l = (b_{l,1}(x), \ldots, b_{l,K_l}(x))$ is the vector of the K_l basis functions f_L is composed of and $\boldsymbol{\alpha}'_l = (\alpha_{l,1}, \ldots, \alpha_{l,K_l})$, is the vector of associated coefficients, and the ϵ_i are independent and identically distributed random variables such that $\mathrm{E}(\epsilon_i) = 0$. The cubic regression spline basis of $f_l(x_l)$ is defined by a set of K_l knots, $l = 1, \ldots, L$ spread evenly through the regressor variables domain. Thus, the nonlinear regression problem is split into several small linear regression problems using a set of transformations of the input variable(s), allowing the data to decide which transformations are important. Any identifiability constraints are imposed on the model before fitting ($f_1, \ldots, f_L$ are each only estimable to within an additive constant), and the additive model can be estimated by penalised least squares in the same way as used in the simple univariate model. The details of the penalised least squares estimation can be found in [15].

We assume **any structural relationship** is either approximated by an additive model of cubic regression splines, $f_{(\bullet)}(\mathcal{V}_l; \boldsymbol{\alpha}_{hl}, \mathbf{b}_{hl}, K_{hl})$, where $\boldsymbol{\alpha}'_{hl} = (\alpha_{h,l,1}, \ldots, \alpha_{h,l,K_{hl}-1})$ is the vector of coefficients associated to the piecewise basis functions $\mathbf{b}_{hl} = (b_{l1}(\mathcal{V}_h), \ldots, b_{l,K_{hl}-1}(\mathcal{V}_h))$, a linear relationship or a combination of the two:

$$\mathcal{V}_h = \sum_{l \in \mathcal{V}_h^{pred}} f_l(\mathcal{V}_l; \boldsymbol{\alpha}_{hl}, \mathbf{b}_{hl}, K_{hl}) + \epsilon_h, \tag{10}$$

or

$$\mathcal{V}_h = \sum_{l \in \mathcal{V}_h^{pred}} \beta_{hl} \mathcal{V}_l + \epsilon_h, \tag{11}$$

or

$$\mathcal{V}_h = \sum_{l \in \mathcal{V}_h^{pred_1}} \beta_{hl} \mathcal{V}_l + \sum_{l \in \mathcal{V}_h^{pred_2}} f_l(\mathcal{V}_l; \boldsymbol{\alpha}_{hl}, \mathbf{b}_{hl}, K_{hl}) + \epsilon_h, \tag{12}$$

where $h = \{\mathcal{V}_h : h \in \text{model's endogenous latent variables}\}$ and $\mathcal{V}_h^{pred}$ is the set of all latent variable h's predecessor latent variables defined in Eq (20), $\mathcal{V}_h^{pred_1}$ is the set of all latent variable h's predecessor latent variables whose partial relationship with $\mathcal{V}_h$ is assumed to be linear (turning $f_l(\mathcal{V}_l; \bullet)$ in the identity function), $f_l(\mathcal{V}_l; \bullet) = \mathcal{V}_l)$, $\mathcal{V}_h^{pred_2}$ is the set of all latent variable h's predecessor latent variables whose partial relationship with $\mathcal{V}_h$ is assumed to be nonlinear, $f_l(\bullet; \bullet)$ is defined above and ϵ_h are error terms such that $\mathrm{E}(\epsilon_h) = 0$. Thus, any endogenous latent variable is a linear combination of its predecessor latent variables or its piecewise transformations as determined by f, a cubic regression spline, or a combination of the two.

Step 1: Outer approximation of the latent variables scores: Outer proxies of the latent variables, $\hat{\xi}^{outer}$, are calculated as linear combinations of their respective indicators. These outer proxies are standardised; i.e. they have a mean of 0 and a standard deviation of 1.The weights of the linear combinations result from step 4 of the previous iteration. When the algorithm is initialised, and no weights are available yet, any arbitrary non trivial linear combination of indicators can serve as an outer proxy of a latent variable [5].

Calculating outer proxies of latent variable scores: Outer proxies of the latent variables, ξ_j^0, are calculated as linear combinations of their respective indicators. The weights of the linear combinations result from step 4 of the previous iteration or are manually initialised. For each nonlinear term, a new proxy is created as the element-wise transformation of the respective outer estimates [6].

The latent variable outer proxies are estimated as a weighted sum of their respective indicators:

$$\widehat{\boldsymbol{\mathcal{V}}}^{outer} = \mathbf{X}\widehat{\mathbf{W}} \tag{13}$$

To kick-off the algorithm, the outer proxies of the latent variable scores are initialised from Eq (13) by setting the $\widehat{\mathbf{W}} = \mathbf{M}$.

To ensure the identification of the weights they need to be normalised. This normalisation is typically can be done using two different methods. The first is ensuring that after computing the outer proxies, $\widehat{\boldsymbol{\mathcal{V}}}^{outer}$ in Eq (13), the weights of the indicators associated to each latent variable h, $h = 1, \ldots, H$, sum to 1, $\sum_{p=1}^{P_h} w_{ph} = 1$. The second fixes the variance of each proxy to one, i.e., $\mathbf{w}_h' \mathbf{R}_h \mathbf{w}_h = 1$, where $\mathbf{R}_h$ is the empirical correlation matrix of these manifest variables or indicators of the block h (assuming all the manifest variables are scale to zero mean and unit variance).

Assuming all the manifest variables, x_{ph}, $p = 1, \ldots, P_h$, $h = 1, \ldots, H$, are scaled ($\mathrm{E}(x_{ph}) = 0$ and $\mathrm{Var}(x_{ph}) = 1$), the latent variables are also centred (at 0), but must be scaled to have unit variance:

$$\hat{\mathcal{V}}_h^{outer} = \frac{\hat{\mathcal{V}}_h^{outer}}{\sqrt{\mathrm{Var}(\hat{\mathcal{V}}_h^{outer})}}, h = 1, \ldots, H, \tag{14}$$

where $\mathrm{Var}(\hat{\mathcal{V}}_h^{outer})$ is the empirical variance of the outer proxy h, $h = 1, \ldots, H$. Thus, the matrix of outer proxies is obtained $\widehat{\boldsymbol{\mathcal{V}}}^{outer} = (\hat{\boldsymbol{\xi}}_1^{outer}, \ldots, \hat{\boldsymbol{\xi}}_h^{outer}, \hat{\boldsymbol{\eta}}_{h+1}^{outer}, \ldots, \hat{\boldsymbol{\eta}}_H^{outer})$.

The $n \times H$ matrix $\widehat{\boldsymbol{\mathcal{V}}}^{outer}$ of outer approximation of latent variable proxies resulting from step 1 ((Eq 13) and (Eq 14)) is augmented to a new matrix $\widehat{\boldsymbol{\mathcal{V}}}_{Aug}^{outer}$ whose number of columns depends upon the specification of the structural model relationships.

The matrix $\widehat{\boldsymbol{\mathcal{V}}}_{Aug}^{outer}$, in Eq (13), has H columns given by

$$\hat{\boldsymbol{\mathcal{V}}}_h^{outer\prime} = (\hat{\mathcal{V}}_{1h}^{outer}, \ldots, \hat{\mathcal{V}}_{nh}^{outer}), h = 1, \ldots, H,$$

where $\widehat{\mathcal{V}}_{ih}^{outer}$ is the estimated outer proxy of ith observation resulting from step 1, as usual, but it is expanded to accommodate as many columns as necessary to account for the nonlinear relationships that are assumed to be nonlinear. Thus, for each partial nonlinear relationship approximated by a cubic regression spline with K_h knots, the matrix $\widehat{\boldsymbol{\mathcal{V}}}^{outer}$ contains $K_h - 1$ additional columns,

$$\begin{aligned}
\mathbf{b}_1(\hat{\boldsymbol{\mathcal{V}}}_h^{outer})' &= (b_1(\hat{\mathcal{V}}_{1h}^{outer}), \ldots, b_1(\hat{\mathcal{V}}_{nh}^{outer})), \\
\mathbf{b}_2(\hat{\boldsymbol{\mathcal{V}}}_h^{outer})' &= (b_2(\hat{\mathcal{V}}_{2h}^{outer}), \ldots, b_2(\hat{\mathcal{V}}_{nh}^{outer})), \\
\ldots &= \ldots \\
\mathbf{b}_{K_h-1}(\hat{\boldsymbol{\mathcal{V}}}_h^{outer})' &= (b_{K_h-1}(\hat{\mathcal{V}}_{1h}^{outer}), \ldots, b_{K_h-1}(\hat{\mathcal{V}}_{nh}^{outer})), h = 1, \ldots, H,
\end{aligned}$$

where $b_k(\widehat{\mathcal{V}}_{ih}^{outer})$ denotes the basis of piecewise linear transformations of the estimated outer proxies, $\widehat{\mathcal{V}}_{ih}^{outer}$, $i = 1, \ldots, n$, $h = 1, \ldots, H$ resulting as usual from step 1. The piecewise linear transformations are determined entirely by the locations at which the linear pieces join up, and the knots $\{\mathcal{V}_{hk}^* : h = 1, \ldots, H,\ k = 1, \ldots, K_h\}$, assuming $\mathcal{V}_{h,k}^* > \mathcal{V}_{h,(k-1)}^*$.

The columns of matrix $\widehat{\boldsymbol{\mathcal{V}}}_{Aug}^{outer}$ should be scaled to a mean of zero and unit variance, as in Eq (14). The identifiability restriction mentioned at the end of the previous section plays a role here. Indeed, K_h must be chosen up to a maximum, such that $(\max(K_h) - 1) \times H < n$, $h = 1, \ldots, H$, where n is the effective sample size.

It is worth mentioning that the level of smoothness for the model, determined by the basis dimension K_h, may be subjective. In fact, as [15] points out, the basis dimension can be set slightly larger than what is believed to be necessary, as the model's smoothness is regulated by adding a penalty to the least squares fitting objective function.

The implementation referred to in **??** allows the user to set the value of K_h. If the user does not have a clue about the dimension of the basis to be used, we recommend to use the value $K_h = 10$, the default value used by `mgcv` package or adjust it to the maximum possible value (fewer than 10) if the sample size does not allow such dimension. The author's software implementation contains a diagnosis function that allows the user

to adjust K_h in a interactive fashion. It even allows the user to downscale a structural partial relationship initially approximated by a smoothing function with a dimension of the basis $K_h \geq 3$ to a linear form. More details on the cubic regression spline piecewise basis functions, $b_k(\hat{\mathcal{V}}_{ih})$, $k = 1, \ldots, K_h - 1$, $i = 1, \ldots, n$, $h = 1, \ldots, H$ are given in [15].

For the sake of a comprehensive understanding of this novel inner weighting scheme, an illustration using the structural model of Fig 1 follows. Let us assume the structural model depicted in Fig 1 is described by the following set of structural equations:

$$\begin{aligned}
\xi_1 &= \xi_1 + 0\\
\eta_2 &= \beta_2\xi_1 + \epsilon_1\\
\eta_3 &= f(\xi_1; \boldsymbol{\alpha}_{\xi_1}, \mathbf{b}_{\xi_1}; K_{\xi_1}) + \beta_4\eta_2 + \epsilon_2\\
\eta_4 &= \beta_1\xi_1 + f(\eta_3; \boldsymbol{\alpha}_{\eta_3}, \mathbf{b}_{\eta_3}; K_{\eta_3}) + \epsilon_3.
\end{aligned} \tag{15}$$

We are assuming η_2 is a linear function of ξ_1, η_3 is a function of two partial relationships, one is a smooth function of ξ_1 (a cubic regression spline with a dimension of the basis given by K_{ξ_1}) and the other is a linear function of η_2, and η_4 is another function of two partial relationships, one is a linear function of ξ_1 and the other is a smooth function of η_3 (a cubic regression spline with a dimension of the basis given by K_{ξ_1}).

The $(n \times (4 + (K_{\xi_1} - 1) + (K_{\eta_3} - 1)))$ outer proxies augmented matrix $\hat{\boldsymbol{\mathcal{V}}}^{outer}_{Aug}$ is given by

$$\begin{pmatrix}
\hat{\xi}_{11} & \hat{\eta}_{12} & \hat{\eta}_{13} & \hat{\eta}_{14} & b_1(\hat{\xi}_{11}) & \cdots & b_{K_{\xi_1}-1}(\hat{\xi}_{111}) & b_1(\hat{\eta}_{13}) & \cdots & b_{K_{\eta_3}-1}(\hat{\eta}_{13})\\
\hat{\xi}_{21} & \hat{\eta}_{22} & \hat{\eta}_{23} & \hat{\eta}_{24} & b_1(\hat{\xi}_{21}) & \cdots & b_{K_{\xi_1}-1}(\hat{\xi}_{121}) & b_1(\hat{\eta}_{23}) & \cdots & b_{K_{\eta_3}-1}(\hat{\eta}_{23})\\
\vdots & \vdots & \vdots & \vdots & \vdots & \ddots & \vdots & \vdots & \ddots & \vdots\\
\hat{\xi}_{n1} & \hat{\eta}_{n2} & \hat{\eta}_{n3} & \hat{\eta}_{n4} & b_1(\hat{\xi}_{n1}) & \cdots & b_{K_{\xi_1}-1}(\hat{\xi}_{1n1}) & b_1(\hat{\eta}_{n3}) & \cdots & b_{K_{\eta_3}-1}(\hat{\eta}_{n3})
\end{pmatrix} \tag{16}$$

Step 2: Estimation of the inner weights: Inner weights are calculated for each latent variable in order to reflect how strongly the other latent variables are connected to it. There are three schemes available for determining the inner weights. [8] originally proposed the centroid scheme. Later, [2] developed the factor weighting and path weighting schemes. The centroid scheme uses the sign of the correlations between a latent variable or, more precisely, the outer proxy and its adjacent latent variables; the factor weighting scheme uses the correlations. The path weighting scheme pays tribute to the arrow orientations in the path model. The weights of those latent variables that explain the focal latent variable are set to the regression coefficients stemming from a regression of the focal latent variable (regressant) on its latent regressor variables. The weights of those latent variables, which are explained by the focal latent variable, are determined in a similar manner as in the factor weighting scheme. Regardless of the weighting scheme, a weight of zero is assigned to all non-adjacent latent variables [5].

Estimating inner weights: For each outer proxy, inner weights are calculated to reflect how strongly the proxies of the other latent variables are connected to it. Several inner weighting schemes are available. Wold (1982) originally proposed that the sign should be used of the correlations between a latent variable and its adjacent latent variables (which is the so-called centroid scheme). Alternatives are the factor weighting scheme and the path weighting scheme [2]. *Regardless of the weighting scheme, a weight of zero is assigned to all non-adjacent latent variables.* [6].

For any the structural model, the adjacency matrix Eq (2), $\mathbf{D}$ (referring to model in Fig 1) accounts for the directionality. For every $d_{ij} = 1$, there is an link between from node i and node j. We could also say, the columns indicate the *successors*, whereas the rows indicate the *predecessors*. As we will see, the adjacency matrix $\mathbf{D}$ facilitates the calculation of the inner weights. For all the weighting schemes, each latent variable is constructed as a weighted sum of the latent variables it is related with. The weighting schemes differ in the way the relation is defined. Furthermore, let us denote $\hat{\mathbf{R}} = \text{Cor}(\hat{\boldsymbol{\mathcal{V}}}^{outer})$, the empirical correlation matrix for the latent variables proxies resulting from the outer estimation, $r_{ij} = \text{Cor}(\hat{\mathcal{V}}_i^{outer}, \hat{\mathcal{V}}_j^{outer})$, $i, j = 1, ...H$, and $\mathbf{C} = \mathbf{D} + \mathbf{D}'$ a symmetrical matrix indicating whether two latent variables are neighbours, $c_{ij} = 1$, and $c_{ij} = 0$ otherwise, $i, j = 1, \ldots, H$.

If the *centroid weighting* scheme is used, the elements of the inner weights matrix, $\mathbf{E}$, are given by

$$e_{ij} = \begin{cases} sign(r_{ij}), & \text{for } c_{ij} = 1, \\ 0, & \text{otherwise} \end{cases}, \ i, j = 1, \ldots, H. \tag{17}$$

If the *factorial weighting* scheme is used, the elements of the inner weights matrix, $\mathbf{E}$, are given by

$$e_{ij} = \begin{cases} r_{ij}, & \text{for } c_{ij} = 1, \\ 0, & \text{otherwise} \end{cases}, \ i, j = 1, \ldots, H. \tag{18}$$

For the *path weighting* scheme the predecessors and successors of a latent variable play a different role in the relation. Let us define the successor set of a node h as the set of latent variables variable h impacts on, represented by the vector

$$\boldsymbol{\mathcal{V}}_h^{succ'} = \{\mathcal{V}_l : l \in \text{successors of } \mathcal{V}_h\}. \tag{19}$$

Likewise, a predecessor set of a node h is the set of latent variables impacting on h, denoted by the vector

$$\boldsymbol{\mathcal{V}}_h^{pred'} = \{\mathcal{V}_l : l \in \text{predecessors of } \mathcal{V}_h\}. \tag{20}$$

.

The relation for one specific latent variable $\mathcal{V}_h$ with its successors is determined by their correlation, $\text{Cor}(\hat{\mathcal{V}}_h, \widehat{\boldsymbol{\mathcal{V}}}_h^{succ})$. For the predecessors it is determined by a multiple regression

$$\hat{\mathcal{V}}_h = \widehat{\boldsymbol{\mathcal{V}}}_h^{pred\prime} \boldsymbol{\gamma}_h + \zeta_h, \ \text{E}(\zeta_h) = 0, \ h = 1, \ldots, H. \tag{21}$$

Therefore, the elements of matrix $\mathbf{E}$ are:

$$e_{ij} = \begin{cases} \hat{\boldsymbol{\gamma}}_h, & \text{for } j \in \mathcal{V}_h^{pred}, \\ \text{Cor}(\hat{\mathcal{V}}_h, \widehat{\boldsymbol{\mathcal{V}}}_h^{succ}), & \text{for } j \in \mathcal{V}_h^{succ}, \ i, j = 1, \ldots, H \\ 0, & \text{otherwise} \end{cases} . \tag{22}$$

It is noteworthy to mention that, according to [16], all of three described schemes yield similar results.

For the hybrid approach, in *smooth weighting* scheme, the inner weights were also determined for each piecewise linear term described in Eq (16) connected to a latent variable. As in *path weighting* scheme, the endogenous and exogenous latent variables have different treatments.

The inner weights matrix, $\mathbf{E}^{Aug}$, will also be augmented. It will contain as many rows as columns of $\widehat{\boldsymbol{\mathcal{V}}}_{Aug}^{outer}$ and H columns, the number of latent variables.

Every endogenous latent variable is given by one of the models in Eq (10), Eq (11) or Eq (12).

Every exogenous latent variable has a set other latent variables it impacts on, its successors defined in Eq (19). The impact on its successors may be through it own direct impact and/or through a set of piecewise linear terms as determined in Eq (10), Eq (11) or Eq (12).

The elements of the columns of $\mathbf{E}^{Aug}$ regarding exogenous latent variables ($\boldsymbol{\xi}_h$) are given as follows:

1. $\text{Cor}(\hat{\xi}_h, \hat{\mathcal{V}}_l)$, if ξ_h is exogenous and impacts directly on $\mathcal{V}_l \in \boldsymbol{\xi}_h^{succ}$;
2. $\text{Cor}(\hat{\xi}_h, \mathbf{b}_{l,1}(\hat{\mathcal{V}}_l)), ..., \text{Cor}(\hat{\xi}_h, \mathbf{b}_{l,K_h-1}(\hat{\mathcal{V}}_l))$ if ξ_h is exogenous and impacts on $\mathcal{V}_l \in \boldsymbol{\xi}_h^{succ}$ through its piecewise linear transformations;
3. 0, otherwise,

where $\text{Cor}(\hat{\xi}_h, b_{l,1}(\hat{\mathcal{V}}_l)), ..., \text{Cor}(\hat{\xi}_h, b_{l,K_h-1}(\hat{\mathcal{V}}_l)$ are the empirical correlations between $\hat{\xi}_h$ and the piecewise transformations of $\mathcal{V}_l$ as defined in Eq (10) or Eq (12) and $\text{Cor}(\hat{\xi}_h, \hat{\mathcal{V}}_l)$ is the empirical correlation between $\hat{\xi}_h$ and $\hat{\mathcal{V}}_l$.

The elements of the columns of $\mathbf{E}^{Aug}$ regarding endogenous latent variables ($\boldsymbol{\eta}_h$) are given as follows:

1. β_l if $\mathcal{V}_l \in \boldsymbol{\eta}_h^{pred}$ and $\mathcal{V}_l$ impacts directly on η_h;
2. $\alpha_{l,1}, \ldots, \alpha_{l,(K_l-1)}$ if $\mathcal{V}_l \in \boldsymbol{\eta}_h^{pred}$ and $\mathcal{V}_l$ impacts on η_h through its piecewise linear transformations;
3. 0, otherwise,

where β_l is the coefficient of the regression as defined in Eq (11) and $\alpha_{l,1}, \ldots, \alpha_{l,(K_l-1)}$ are the regression coefficients associated to piecewise transformations of $\mathcal{V}_l$, $b_{l,1}(\hat{\mathcal{V}}_l), \ldots, b_{l,K_h-1}(\hat{\mathcal{V}}_l)$ as defined in Eq (10) or Eq (12).

Again, let us assume the structural model depicted in Fig 1 is described by the set of structural equations in Eq (15). An illustration of $\widehat{\mathbf{E}}_{Aug}$ using the structural model of Fig 1 follows. The $(4+(K_{\xi_1}-1)+(K_{\eta_3}-1))\times 4$ matrix of augmented inner weights of the structural model depicted in Fig 1 is:

$$\widehat{\mathbf{E}}_{Aug} = \begin{array}{c|cccc}
\text{Columns of } \widehat{\boldsymbol{\mathcal{V}}}_{Aug}^{outer} & \xi_1 & \eta_2 & \eta_3 & \eta_4 \\
\hline
\xi_1 & 0 & \beta_2 & 0 & \beta_1 \\
\eta_2 & \text{Cor}(\hat{\xi}_1, \hat{\eta}_2) & 0 & \beta_4 & 0 \\
\eta_3 & 0 & 0 & 0 & 0 \\
\eta_4 & 0 & 0 & 0 & 0 \\
\hline
b_1(\hat{\xi}_1) & 0 & 0 & \alpha_{\xi_1,1} & 0 \\
\vdots & \vdots & \vdots & \vdots & \vdots \\
b_{K_{\xi_1}-1}(\xi_1) & 0 & 0 & \alpha_{\xi_1,K_{\xi_1}-1} & 0 \\
\hline
b_1(\eta_3) & \text{Cor}(\hat{\xi}_1, b_1(\hat{\eta}_3)) & 0 & 0 & \alpha_{\eta_3,1} \\
\vdots & \vdots & \vdots & \vdots & \vdots \\
b_{K_{\eta_3}-1}(\eta_3) & \text{Cor}(\hat{\xi}_1, b_{K_{\eta_3}-1}(\hat{\eta}_3)) & 0 & 0 & \alpha_{\eta_3,K_{\xi_1}-1}
\end{array}$$

Indeed, η_2 is a linear function of ξ_1, η_3 is a linear function of piecewise transformations of ξ_1 and a linear function of η_2, and η_4 is a linear function ξ_1 and a linear function of piecewise transformations of η_3, and ξ_1 is exogenous and its direct successors are η_2, and it impacts on η_3 and the piecewise transformations of η_3.

Step 3: Inner approximation of the latent variable scores: Inner proxies of the latent variables, ξ_j^{inner}, are calculated as linear combinations of the outer proxies of their respective adjacent latent variables, using the afore-determined inner weights [5].

Calculating inner proxies of latent variable scores: Inner proxies of the latent variables, ξ_j^0, are calculated as linear combinations of their respective adjacent latent variables' outer proxies, using the previously determined inner weights [6].

In the inner approximation, we estimate each latent variable as a weighted sum of its neighbouring latent variables. The weighting depends on the used scheme described above:

$$\widehat{\boldsymbol{\mathcal{V}}}^{inner} = \widehat{\boldsymbol{\mathcal{V}}}^{outer} \widehat{\mathbf{E}}. \tag{23}$$

The estimated inner proxies $\widehat{\boldsymbol{\mathcal{V}}}^{inner} = (\hat{\xi}_1^{inner}, \ldots, \hat{\xi}_h^{inner}, \hat{\eta}_{h+1}^{inner}, \cdots, \hat{\eta}_H^{inner})$ are obtained by scaling the recomputed latent variables proxies to have unit variance:

$$\hat{\mathcal{V}}_h^{inner} = \frac{\hat{\mathcal{V}}_h^{inner}}{\sqrt{\text{Var}(\hat{\mathcal{V}}_h^{inner})}}, h = 1, \ldots, H, \tag{24}$$

where $\text{Var}(\hat{\mathcal{V}}_h^{inner})$ is the empirical variance of the outer proxy h, $h = 1, \ldots, H$.

In the **smoothing-based hybrid approach**, the piecewise basis linear functions of the latent variable outer scores in Eq (16), $b_k(\hat{\xi}_{ih})$, $k = 1, \ldots, K_h$, $i = 1, \ldots, n$ and $h = 1, \ldots, H$ are also used to estimate endogenous latent variables' inner proxies.

In the inner approximation we estimate each latent variable as a weighted sum of its neighbouring latent variables,

$$\widehat{\boldsymbol{\mathcal{V}}}_{Aug}^{inner} = \widehat{\boldsymbol{\mathcal{V}}}_{Aug}^{outer}\widehat{\mathbf{E}}^{Aug}. \tag{25}$$

The inner proxies are scaled to have unit variance:

$$\hat{\mathcal{V}}_h^{inner} = \frac{\hat{\mathcal{V}}_h^{inner}}{\sqrt{\mathrm{Var}(\hat{\mathcal{V}}_h^{inner})}},\ h = 1, \ldots, H, \tag{26}$$

Step 4: Estimation of the outer weights: The outer weights are calculated either as the covariances between the inner proxy of each latent variable and its indicators (in Mode A), or as the regression weights resulting from the ordinary least squares regression of the inner proxy of each latent variable on its indicators (in Mode B) [5].

Mode A: A multivariate regression coefficient with the block of manifest variables as response and the latent variable as the regressor:

$$\hat{\mathbf{w}}_h' = (\hat{\mathcal{V}}_h^{inner\prime}\hat{\mathcal{V}}_h^{inner})^{-1}\hat{\mathcal{V}}_h^{inner\prime}\mathbf{X}_h \tag{27}$$
$$= \mathrm{Cor}(\hat{\mathcal{V}}_h^{inner}, \mathbf{X}_h), h = 1, \ldots, H. \tag{28}$$

Mode B: A multiple regression coefficient with the latent variable as response and its block of manifest variables as regressors:

$$\hat{\mathbf{w}}_h' = (\mathbf{X}_h'\mathbf{X}_h)^{-1}\mathbf{X}_h\hat{\mathcal{V}}_h^{inner} \tag{29}$$
$$= \mathrm{Var}(\mathbf{X}_h)^{-1}\mathrm{Cor}(\mathbf{X}_h, \hat{\mathcal{V}}_h^{inner}), h = 1, \ldots, H. \tag{30}$$

These steps are iterated until the change in outer weights between two consecutive iterations falls below a predefined relative tolerance,

$$\max\left(\frac{w_{ph}^{(i)} - w_{ph}^{(i+1)}}{w_{ph}^{(i+1)}}\right) < T_r,\ p = 1, \ldots, P_h;\ h = 1, \ldots, H, \tag{31}$$

or absolute tolerance,

$$\max\sqrt{\left(w_{ph}^{(i)} - w_{ph}^{(i+1)}\right)^2} < T_a,\ p = 1, \ldots, P_h;\ h = 1, \ldots, H, \tag{32}$$

where i denotes the iteration. The default tolerance used in the authors' software implementation relative tolerance, T_r, is fixed at a very tiny level, 1×10^{-7}, whereas the absolute tolerance, T_a is fixed at 0.001.

Once again, to ensure the identification of the weights they need to be normalised which is done as before, that is, ensuring that after computing the inner weights of the indicators associated to each latent variable h, $h = 1, \ldots, H$, sum to 1, $\sum_{p=1}^{P_h} w_{ph} = 1$, or by fixing the variance of each proxy to one, i.e., $\mathbf{w}_h'\mathbf{R}_h\mathbf{w}_h = 1$, where $\mathbf{R}_h$ is the empirical correlation matrix of these manifest variables or indicators of the block h (assuming all the manifest variables are scale to zero mean and unit variance).

In the **smoothing-based hybrid approach** no additional procedure is required in this step 4 because the piecewise linear terms do not have any assigned manifest variables, as determined by the hybrid approach.

Once convergence has been achieved, the latent variable scores are the outer proxies resulting from the last iteration, which are to estimate the path coefficients. Although other methods may be used, the path

coefficients can be estimated by ordinary least squares (OLS), according to the structural model (1). For each latent variable $\mathcal{V}_h$, $h = 1, \ldots, H$, the path coefficient is the regression coefficient on its predecessor set $\boldsymbol{\mathcal{V}}_h^{pred}$:

$$\boldsymbol{\beta}_h = (\widehat{\boldsymbol{\mathcal{V}}}_h^{pred'} \widehat{\boldsymbol{\mathcal{V}}}_h^{pred})^{-1} \widehat{\boldsymbol{\mathcal{V}}}_h^{pred'} \hat{\mathcal{V}}_h \tag{33}$$

$$= \mathrm{Cor}(\widehat{\boldsymbol{\mathcal{V}}}_h^{pred}, \widehat{\boldsymbol{\mathcal{V}}}_h^{pred})^{-1} \mathrm{Cor}(\widehat{\boldsymbol{\mathcal{V}}}_h^{pred}, \hat{\mathcal{V}}_h). \tag{34}$$

As has been described, the PLS-PM algorithm is closely related to OLS estimation techniques. It focuses on predicting a specific set of hypothesised linear relationships that maximise the explained variance in the dependent variables (endogenous latent variables), similar to OLS regression models. This feature makes PLS-PM a suitable technique for prediction, although its potential for explanation should not be overlooked ([17]). Nevertheless, PLS-PM does not aim to optimise a global scalar function. The focus of PLS-PM is the discrepancy between the observed (in the case of manifest variables) or approximated (in the case of latent variables) values of the dependent variables and the values predicted by the model [4]. Consequently, researchers using PLS-PM usually rely on quality indices, such as R^2, cross-validated communality, or redundancy, to judge the quality of the model.

However, it is worth considering whether the traditional assumption of linear relationships between latent variables and observed indicators is sufficient to capture all relationships in every case. This is especially true, given that some theories suggest that nonlinear relationships may exist in some situations (for example, [18]). If the answer to this question is no, it is unclear what alternative methods could be used to account for measurement errors in manifest variables while avoiding contamination of latent variable scores and ultimately affecting structural path coefficients [19,20].

In the **smoothing-based hybrid approach**, the structural relationships in Eq (10), Eq (11) and Eq (12) are estimated using the factor scores. Estimation is conducted using penalised least squares for all the structural relationships involving at least one regression cubic spline.

This **smoothing-based hybrid approach** comprising the novel inner *smooth weighting* scheme, the changes in steps 1, 2 and 3, and the new approach to estimate structural relationships is hereafter denoted by **PLSs-PM**.

Let $\hat{\boldsymbol{\beta}}$ be the estimator of $\boldsymbol{\beta}$, the transition matrix for the structural model:

$$\hat{\beta}_{hj} = \begin{cases} \beta_{hj}, & j \in \boldsymbol{\mathcal{V}}_h^{pred} \\ 0, & \text{otherwise} \end{cases} \quad h = 1, \ldots, H. \tag{35}$$

The impact of all model latent variables on a single latent variable is given by the matrix of total effects $\hat{\boldsymbol{\tau}}$. It can be obtained as the sum of the 1 to H step transition matrices:

$$\hat{\boldsymbol{\tau}} = \sum_{h=1}^{H} \hat{\boldsymbol{\beta}}^h = \underbrace{\hat{\boldsymbol{\beta}} \times \hat{\boldsymbol{\beta}} \times \cdots \times \hat{\boldsymbol{\beta}}}_{h \text{ times}}. \tag{36}$$

For example $\hat{\boldsymbol{\beta}}^2$ contains all the indirect effects mediated by only one latent variable. The cross and outer loadings are estimated as:

$$\hat{\boldsymbol{\Lambda}}^{cross} = \mathrm{Cor}(\mathbf{X}, \boldsymbol{\mathcal{V}}^{\hat{outer}}), \tag{37}$$

$$\hat{\lambda}_{ph}^{outer} = \begin{cases} \hat{\lambda}_{ph}^{cross}, & m_{ph} = 1 \\ 0 & \text{otherwise,} \end{cases} \tag{38}$$

where m_{ph}, $p = 1, \ldots, P_h$, $h = 1, \ldots, H$ are the element of the adjacency matrix of the outer model $\mathbf{M}$. In the particular case of the model depicted in Fig 1, $\mathbf{M}$ is given in Eq (7).

In 2015, to address the bias in structural regression parameter estimates caused by the inclusion of measurement errors in composites, [19] and [20] developed the consistent PLS-PM (PLSc). This was done by utilising

PLS-PM indicator weights for mode A weighting and reflective indicators to establish a consistent estimate of the reliability of a composite variable:

$$\rho_A(\hat{\mathcal{V}}_h) = (\hat{\mathbf{w}}'_h\hat{\mathbf{w}}_h)^2 \frac{\hat{\mathbf{w}}'_h(\mathbf{S}_h - \mathrm{diag}(\mathbf{S}_h))\hat{\mathbf{w}}_h}{\hat{\mathbf{w}}'_h(\hat{\mathbf{w}}_h\hat{\mathbf{w}}'_h - \mathrm{diag}(\hat{\mathbf{w}}_h\hat{\mathbf{w}}'_h))\hat{\mathbf{w}}_h}, \quad (39)$$

where $\hat{\mathbf{w}}_h$ is the vector of estimated outer weights of $\mathcal{V}_h$ indicators' block obtained in Step 3 and $\mathbf{S}_h$ is its empirical correlation matrix, h, $h = 1, \ldots, H$.

In the first step, the traditional PLS-PM algorithm is applied. This computes the outer latent variable proxies, $\hat{\mathcal{V}}_h^{outer}$, and outer weight vectors, $\hat{\mathbf{w}}_h$ for each latent variable proxy, $h = 1, \ldots, H$. The biased estimate of the latent variable correlation between latent variables $\hat{\mathcal{V}}_h$ and $\hat{\mathcal{V}}_{h'}$ is then corrected using the well-known correction for attenuation introduced by [21]:

$$\mathrm{Cor}(\hat{\mathcal{V}}_i, \hat{\mathcal{V}}_j) = \frac{\mathrm{Cor}^*(\hat{\mathcal{V}}_i, \hat{\mathcal{V}}_j)}{\sqrt{\rho_A(\hat{\mathcal{V}}_i)\rho_A(\hat{\mathcal{V}}_j)}}, \; i, h = 1, \ldots, H, \quad (40)$$

where $\mathrm{Cor}^*(\hat{\mathcal{V}}_i, \hat{\mathcal{V}}_j)$ is the empirical correlation matrix between $\hat{\mathcal{V}}_i^{outer}$'s and $\hat{\mathcal{V}}_j^{outer}$'s outer proxies.

Finally, consistent estimates of loadings can be derived using $\rho_A(\hat{\mathcal{V}}_h)$ and $\hat{\mathbf{w}}_h$.

[19] conducted initial simulation studies that showed that the estimates provided by PLSc were close to that of the maximum likelihood (ML) estimator, with little bias and comparable precision for structural parameters but less precision for item loadings. This was later confirmed by a comparison using a real dataset, where PLSc estimates were found to be close to those obtained through covariance-based estimates [22]. However, not all studies have found similar encouraging results. [23] and [24] found that PLSc loading estimates were less precise and also more biased than traditional maximum likelihood estimates. They also showed that PLSc tends to overestimate small correlations and underestimate large correlations, which may be due to the capitalisation on chance and overestimation of reliability [25]. Additionally, PLSc produces more inaccurate results, and most recently, [26] (pp. 334) argue that there is no clear advantage of PLSc over using unweighted scales and disattenuating with a coefficient of (Cronbach's alpha). [20] (pp. 309) noted that, *among the consistent techniques, PLSc typically had the lowest statistical power.*

1. Wold HOA. Nonlinear estimation by iterative least squares procedures. In: David FN, Neyman J, editors. Research papers in statistics. Wiley; 1966.

2. Lohmöller JB. Latent variable path modeling with partial least squares. Physica Heidelberg; 1989. doi:10.1007/978-3-642-52512-4

3. Tenenhaus M, Vinzi VE, Chatelin YM, Lauro C. PLS path modeling. Computational Statistics & Data Analysis. 2005;48: 159–205. doi:10.1016/j.csda.2004.03.005

4. Hair JF, Hult GTM, Ringle CM, Sarstedt M, Danks NP, Ray S. An introduction to structural equation modeling. Partial least squares structural equation modeling (PLS-SEM) using r: A workbook. Cham: Springer International Publishing; 2021. pp. 1–29. doi:10.1007/978-3-030-80519-7_1

5. Henseler J, Ringle CM, Sinkovics RR. The use of partial least squares path modeling in international marketing. In: Sinkovics RR, Ghauri PN, editors. New challenges to international marketing (advances in international marketing, vol 20). Emerald Group Publishing Limited; 2009. pp. 277–319. Available: 10.1108/S1474-7979(2009)0000020014

6. Henseler J, Fassott D, Dijkstra TK, Wilson B. Analysing quadratic effects of formative constructs by means of variance-based structural equation modelling. European Journal of Information Systems. 2012;21: 99–112. doi:10.1057/ejis.2011.36

7. Schamberger T, Schuberth F, Henseler J, Dijkstra TK. Robust partial least squares path modeling. Behaviormetrika. 2020;47: 307–334. doi:10.1007/s41237-019-00088-2

8. Wold HOA. Soft modeling: The basic design and some extensions. In: Joreskog KG, Wold HOA, editors. Systems under indirect observations: Part II. Amsterdam: North-Holland; 1982. pp. 1–54.

9. Wahba G. Smoothing splines. In: Lovric M, editor. International encyclopedia of statistical science. Springer; 2011. pp. 4277–4415. doi:10.1007/978-3-642-04898-2_527

10. Akhrif R, Delgado-Márquez E, Kouibia A, Pasadas M. Economic statistical splicing data using smoothing quadratic splines. In: Barrera D, Remogna S, Sbibih D, editors. Mathematical and computational methods for modelling, approximation and simulation. Cham: Springer International Publishing; 2022. pp. 163–179.

11. Mullah M, Hanley JA, Benedetti A. LASSO type penalized spline regression for binary data. BMC Medical Research Methodology. 2021;21. doi:10.1186/s12874-021-01234-9

12. Irizarry RA. Choosing smoothness parameters for smoothing splines by minimizing and estimate of risk. Johns Hopkins University, Department of Biostatistics Working Papers; 2004 Feb. Report No.: Working Paper 30.

13. Early JJ, Sykulski AM. Smoothing and interpolating noisy GPS data with smoothing splines. Journal of Atmospheric and Oceanic Technology. 2020;37: 449–465. doi:10.1175/JTECH-D-19-0087.1

14. Utreras FI. Recent results on multivariate smoothing splines. In: W. Haußmann W, Jetter K, editors. Multivariate approximation and interpolation, proceedings of an international workshop held at the university of duisburg, august 14-18, 1989. Birkhäuser, Basel; 1990. pp. 239–322. doi:10.1007/978-3-0348-5685-0_23

15. Wood SN. Generalized additive models: An introduction with r. 2nd ed. Chapman; Hall/CRC; 2017.

16. Noonan R, Wold H. PLS path modeling with indirectly observed variables: A comparison of alternative estimates for the latent variable. In: Jöreskog KG, Wold H, editors. Systems under indirect observation: Causality, structure, prediction part II. 1982. pp. 75–79.

17. Hair JF, Ringle CM, Sarstedt M. PLS-SEM: Indeed a silver bullet. Journal of Marketing Theory and Practice. 2011;19: 139–152. doi:10.2753/MTP1069-6679190202

18. Tuu H, Olsen S. Nonlinear effects between satisfaction and loyalty: An empirical study of different conceptual relationships. Journal of Targeting, Measurement and Analysis for Marketing. 2010;18: 239–251. doi:10.1057/jt.2010.19

19. Dijkstra TK, Henseler J. Consistent and asymptotically normal PLS estimators for linear structural equations. Computational Statistics & Data Analysis. 2015;81: 10–23. doi:10.1016/j.csda.2014.07.008

20. Dijkstra TK, Henseler J. Consistent partial least squares path modeling. MIS Quarterly. 2015;39: 297–316. doi:10.25300/MISQ/2015/39.2.02

21. Spearman C. The proof and measurement of association between two things. The American Journal of Psychology. 1904;15: 441–471. doi:10.2307/1412159

22. Henseler J. Bridging design and behavioral research with variance-based structural equation modeling. Journal of Advertising. 2017;46: 178–192. doi:10.1080/00913367.2017.1281780

23. Huang W. PLSe: Efficient estimators and tests for partial least squares. PhD thesis, University of California. 2013. Available: http://escholarship.org/uc/item/2cs2g2b0

24. Rönkkö M, McIntosh CN, Antonakis J, Edwards JR. Partial least squares path modeling: Time for some serious second thoughts. Journal of Operations Management. 2016;47-48: 9–27. doi:https://doi.org/10.1016/j.jom.2016.05.002

25. Aguirre-Urreta MII, Rönkkö M, McIntosh CN. A cautionary note on the finite sample behavior of maximal reliability. Psychological methods. 2019;24: 236–252. doi:10.1037/met0000176

26. Yuan K-H, Y. Wen, Tang J. Regression analysis with latent variables by partial least squares and four other composite scores: Consistency, bias and correction. Structural Equation Modeling: A Multidisciplinary Journal. 2020;27: 333–350. doi:10.1080/10705511.2019.1647107
